# Co-evolution of primitive methane-cycling ecosystems and early Earth’s atmosphere and climate

**DOI:** 10.1038/s41467-020-16374-7

**Published:** 2020-06-01

**Authors:** Boris Sauterey, Benjamin Charnay, Antonin Affholder, Stéphane Mazevet, Régis Ferrière

**Affiliations:** 1 Institut de Biologie de l’Ecole Normale Supérieure (IBENS), Université Paris Sciences et Lettres, CNRS, INSERM, 75005 Paris, France; 2 International Center for Interdisciplinary Global Environmental Studies (iGLOBES), CNRS, ENS-PSL University, University of Arizona, Tucson, AZ 85721 USA; 3 Institut de Mécanique Céleste et de Calcul des Ephémérides (IMCCE), Observatoire de Paris, Université PSL, CNRS, Sorbonne Université, Univ. Lille, F-75014 Paris, France; 4 LESIA, Observatoire de Paris, Université PSL, CNRS, Sorbonne Université, Université de Paris, 5 place Jules Janssen, 92195 Meudon, France; 5 Department of Ecology & Evolutionary Biology, University of Arizona, Tucson, AZ 85721 USA

**Keywords:** Element cycles, Palaeoecology, Palaeoceanography

## Abstract

The history of the Earth has been marked by major ecological transitions, driven by metabolic innovation, that radically reshaped the composition of the oceans and atmosphere. The nature and magnitude of the earliest transitions, hundreds of million years before photosynthesis evolved, remain poorly understood. Using a novel ecosystem-planetary model, we find that pre-photosynthetic methane-cycling microbial ecosystems are much less productive than previously thought. In spite of their low productivity, the evolution of methanogenic metabolisms strongly modifies the atmospheric composition, leading to a warmer but less resilient climate. As the abiotic carbon cycle responds, further metabolic evolution (anaerobic methanotrophy) may feed back to the atmosphere and destabilize the climate, triggering a transient global glaciation. Although early metabolic evolution may cause strong climatic instability, a low CO:CH_4_ atmospheric ratio emerges as a robust signature of simple methane-cycling ecosystems on a globally reduced planet such as the late Hadean/early Archean Earth.

## Abstract (web summary)

Biology can profoundly influence the planet’s climate, but over Earth’s long history these effects are poorly constrained. Here the authors show that on early Earth, the evolution of microbes producing and consuming methane likely controlled warming and glacial events, and thus Earth’s habitability

## Introduction

By 3.5 Ga, life had emerged on Earth^1–3^. Astrophysical and geophysical data concur in showing that the planet was habitable 400 My earlier at the very least, and possibly as early as ~4.5 Gya, depending on the occurrence, magnitude, and effect of large asteroid impacts during the Hadean^3^. Early on, Earth’s carbon cycle likely established and maintained temperate climatic conditions^4,5^ in spite of a Sun being 20–25% dimmer than it is today^6^. The earliest microbial ecosystems evolving under these conditions, hundreds of million years before the first anoxygenic phototrophs^7^ became actors of the Archean climate^8^, most likely involved chemolithotrophs (i.e., unicellular organisms that use redox potential as energy source for biomass production) producing methane as a metabolic waste. Phylogenetic analyses^2,7,9,10^ combined with isotopic evidence^11^ and the then-time predominance^12,13^ of the electron donors H_2_ and CO lend weight to a very early origin of H_2_-based methanogens (MG), CO-based autotrophic acetogens (AG), and methanogenic acetotrophs (AT). As CH_4_ built up in the atmosphere, the evolution of anaerobic methanotrophy (MT) may have been favored. Contrary to the modern biosphere, the biomass productivity of such ecosystems was likely low and energy limited, i.e., limited by the availability of electron donors rather than nutrients such as nitrogen, phosphorus, or iron^13,14^. Whether and how the evolution of a primitive biosphere formed by these metabolisms influenced the planetary environment globally is unclear.

Previous studies^8,12,13^ have addressed the productivity of primitive, chemolithotrophic ecosystems and their influence on the young Earth’s equilibrium atmospheric conditions. Such studies relied on equilibrium analyses of the planetary ecosystem; they made strongly simplifying assumptions on the function of chemolithotrophic microbial metabolisms, and did not close the feedback loop linking biological activity, atmospheric composition, and climate. Although these studies showed that primitive biospheres may have had a significant impact on the planet’s early atmosphere and climate, their ability to quantify this impact and estimate the underlying biomass productivity was limited. Furthermore, earlier theory based on equilibrium analyses could not address the coupled dynamics of metabolic evolution and planetary surface conditions, whereby evolutionary changes might trigger significant atmospheric and climatic events and lead to novel steady states. Thus, advancing existing models is needed to generate hypotheses on the history of atmospheric and climatic conditions that metabolic evolutionary innovation may have driven on the early Earth.

**Fig. 1:**
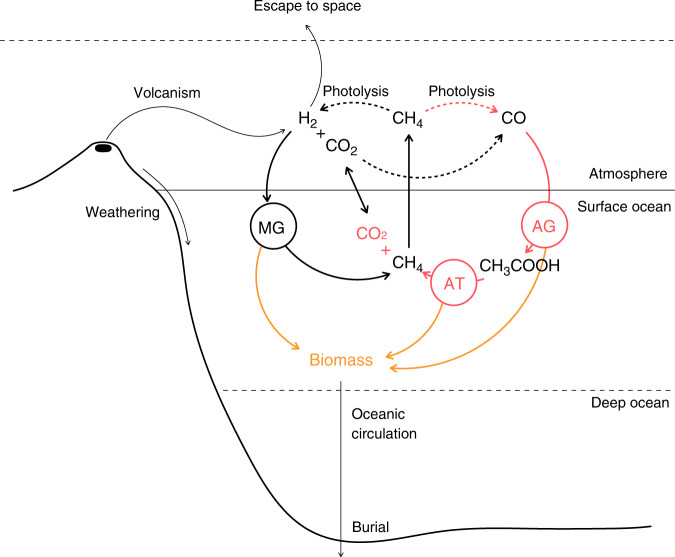
Primitive methanogenic ecosystems. The ecosystem model resolves population abundance (total biomass, yellow arrows) of microbial H_2_-based methanogens (MG), CO-based acetogens (AG) and methanogenic acetotrophs (AT), along with CH_4_, CO, CO_2_, and H_2_ oceanic concentrations and atmospheric mixing ratios. Fluxes directly involved in the MG ecosystem function are indicated with black arrows. Fluxes additionally involved in the AG + AT ecosystem function are indicated in red. Key photochemical reactions are indicated with dotted arrows. The primary source of reducing power (H_2_) is volcanic outgassing. Fluxes across the ocean surface are governed by a stagnant boundary layer model. Rates of H_2_ escape to space and dead biomass burial in deep sediments are constant. Sulfate-based methanotrophs are not represented. See “Methods” for further details.

Here we ask, how constrained was the habitability of late Hadean/early Archean Earth to methane-cycling ecosystems? How productive were these ecosystems and did they have a significant impact on the atmospheric composition and climate? How did their impact change as different metabolisms evolved? To answer these questions, we lay out a new probabilistic modeling framework for an evolving microbial community coupled to early Earth surface geochemistry and climate (Fig. 1, see “Methods” and Supplementary “Results and Discussion” for further details). The mean surface temperature and the composition of the atmosphere and oceans are parameterized using a 3D climate model^4,15^ and a 1D photochemical model^16^, combined with a simple temperature-dependent carbon cycle model^5^. Advancing the previous studies^8,12,13^, this planetary model is coupled dynamically to a biological model of cell population dynamics and evolutionary adaptation, constructed by scaling the intracellular processes of energy acquisition (i.e., catabolism), cell maintenance, and biomass production (i.e., anabolism) up to ecosystem function^17,18^. The biological model is grounded in thermodynamics and based on observations of cell size and temperature kinetic dependencies, widely and robustly shared among modern unicellular organisms^19–23^. Quantitative validation of similar models was obtained from laboratory experiments on anoxic ecosystems in bioreactors^18^.

## Results

### Ecosystems viability

**Fig. 2:**
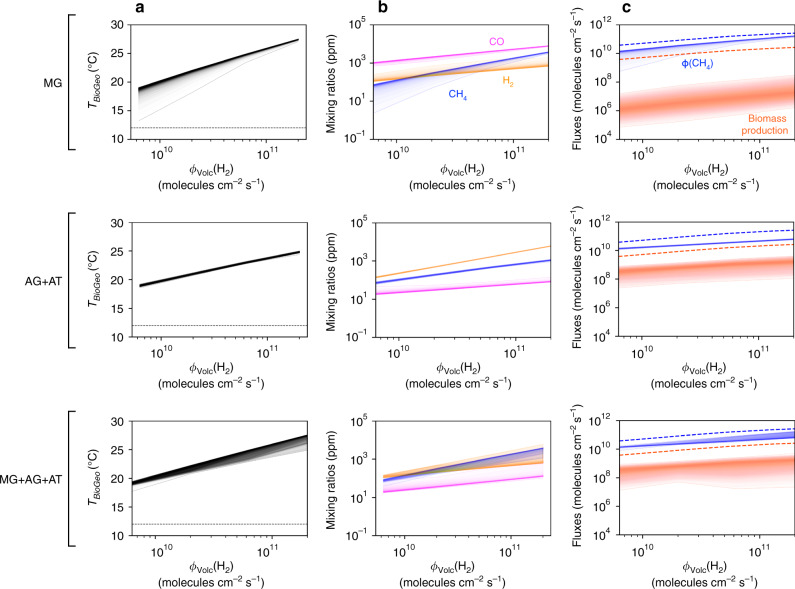
Short-term biological feedback to the atmosphere and climate. Effects are computed as a function of the H2 volcanic outgassing, for each ecosystem composition. MG indicates H_2_-based methanogens (MG). AG+AT indicates CO-based acetogens and methanogenic acetotrophs consortia. MG+AG+AT indicates co-occurring methanogens, acetogens, and acetotrophs. **a** Global surface temperature at ecosystem-climate equilibrium. The dotted line indicates the initial abiotic surface temperature, *T*_Geo_ = 12 °C. **b** Atmospheric composition at ecosystem-climate equilibrium. **c** Biogenic fluxes at ecosystem-climate equilibrium: CH_4_ production and carbon fixation in biomass (in molecules of C cm^−2^ s^−1^). Envelopes represent probability distributions from Monte-Carlo simulations across the biological parameter space, with each layer indicating output frequency ranging from 90 to 51%. Predictions from ref. ^13^ are also shown (dashed) for comparison. See “Methods” and Supplementary Tables 3 and 4 for parameter values.

First, we assess the viability of the methanogenic biospheres (MG, AG + AT, or MG + AG + AT) on an initially cool, lifeless Earth. The initial abiotic surface temperature *T*_Geo_ is assumed to be 12 °C, corresponding to *p*CO_2_ = 2 × 10^5^ ppm, negligible *p*CH_4_, and volcanic outgassing of H_2_, *φ*_volc_(H_2_), ranging from 5 × 10^9^ to 2 × 10^11^ molecules cm^−2^ s^−1^. By performing a Monte-Carlo exploration of the space of biological parameters, we generate posterior distributions of possible life-atmosphere-climate outcomes (Supplementary Figs. 1 and 2), thus providing general insights that do not depend on specific parameterizations of chemolithotrophic metabolisms. We find that all three methanogenic ecosystems are viable (i.e., they can sustain a steady, positive biomass production) in more than 50% of the simulations, regardless of the intensity of H_2_ outgassing (further information on the region of viability in the space of biological parameters is provided in the Supplementary Results and Discussion). Among viable ecosystems, the posterior distributions of the planetary and ecological state variables are peaked and relatively narrow (Fig. 2). Hereafter we will focus on median values to describe model outputs.

### Short-term effects of metabolic evolution

We first consider the direct effects on the early Earth’s atmosphere and climate, on a relatively short timescale of ~10^6^ years, of the transition from the initially cool, lifeless state to a planet populated by one of three methanogenic biospheres, MG, AG + AT, or MG + AG + AT. On such a short timescale, the carbon cycle has a negligible influence on the atmospheric CO_2_. H_2_ is both a metabolic substrate of MG and involved in the photochemical production of CO, the metabolic substrate of AG (Fig. 1). As a consequence, stronger H_2_ volcanic outgassing always enhances biomass production and CH_4_ emission (Fig. 2). The highest CH_4_ emission is achieved by MG ecosystems, with *p*CH_4_ ranging from 80 to 4000 ppm and equilibrium temperature, and *T*_BioGeo_, raised by +7° to +17° (Fig. 2). The environmental impact of AG + AT ecosystems is similar in magnitude, with *p*CH_4_ ranging from 50 to 1000 ppm, and temperature increases of +6° to +15° (Fig. 2).

**Fig. 3:**
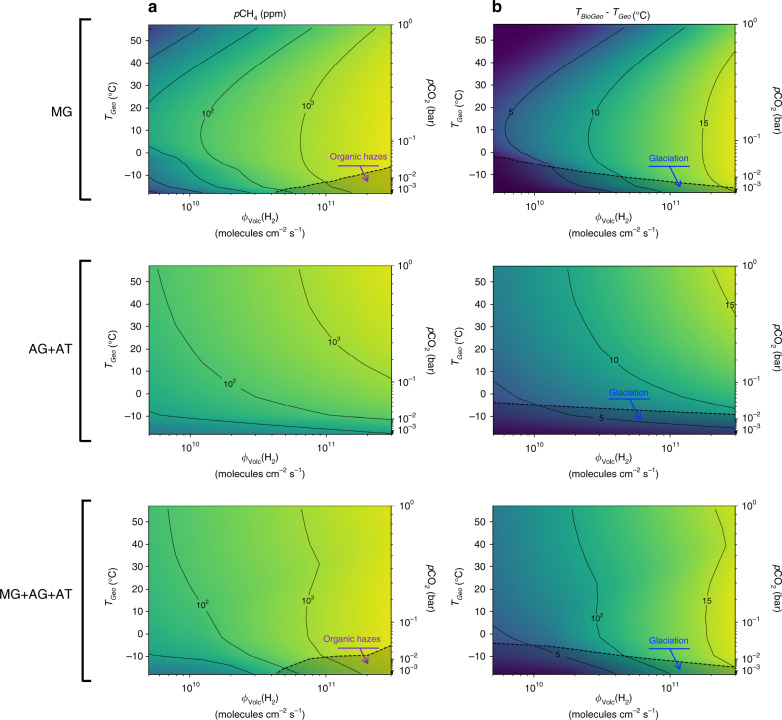
Short-term biological feedback on the atmosphere and climate. Effects are computed as a function of the H_2_ volcanic outgassing and abiotic surface temperature (*T*_Geo_), for each ecosystem composition. MG indicates H_2_-based methanogens. AG+AT indicates CO-based acetogens and methanogenic acetotrophs consortia. MG+AG+AT indicates co-occurring methanogens, acetogens, and acetotrophs. *T*_Geo_ is varied by changing *p*CO_2_ in the climate model. **a** Atmospheric *p*CH_4_ at ecosystem-climate equilibrium. Shaded areas indicate conditions for organic haze formation. **b** Temperature differential between *T*_Geo_ and the global surface temperature reached at ecosystem-climate equilibrium, *T*_BioGeo_. Shaded areas indicate conditions leading to organic haze formation (**a**) and glaciation (**b**). Other parameters are set at their default values (Supplementary Tables 2 and 3).

The planet’s abiotic surface temperature, *T*_Geo_, is likely to have a strong influence on methanogenic activity (see “Methods”). Temperature influences both cell kinetics (metabolisms are slower at lower temperature) and thermodynamics (strong negative effect of high temperatures on MG, less so on AG + AT). *T*_Geo_ also correlates positively with *p*CO_2_ and *p*CO, which are both substrates of methanogenesis. We evaluate the influence of *T*_Geo_ by examining bio-geo environmental feedbacks (i.e., how the change in metabolic activity due to variation in *T*_Geo_ feeds back to climate) for *T*_Geo_ ranging from −18 to 57 °C, which corresponds to *p*CO_2_ ranging from 5 × 10^-4^ to 1 bar (Fig. 3). We ran simulations using a default biological parameterization for which CH_4_ emissions are close to the median predictions described above. Note that we also considered *T*_Geo_ as varying independently of *p*CO_2_, due to e.g. variation in stellar radiation; corresponding results are shown in Supplementary Fig. 3.

If the overall effect of *T*_Geo_ on methanogenic activity is positive, the methanogenic ecosystem is expected to amplify temperature fluctuations driven by external events such as variation in CO_2_ outgassing. In this case, increasing *T*_Geo_ should enhance the biogenic emission of CH_4_, further warming the planet through additional greenhouse effect; decreasing *T*_Geo_ should have the opposite effect. In contrast, if the overall effect of *T*_Geo_ on methanogenic activity is negative, methanogenic ecosystems will buffer temperature variation. For the MG ecosystem (Fig. 3), we find that there is a critical abiotic temperature *T*_Geo_ ≈ 5 °C, almost insensitive to H_2_ outgassing, at which the warming effect of the ecosystem is maximum, from +5° to +17° across the range of H_2_ outgassing rates. The MG ecosystem buffers fluctuations of abiotic temperature above the critical value, whereas it amplifies abiotic temperature fluctuations below the critical value. In contrast, the AG + AT ecosystem always amplifies temperature variation at all abiotic temperatures, and its influence tends to dominate when MG and AG + AT metabolisms co-occur (Fig. 3). Such amplification or buffering of temperature variations can represent up to 33% of the abiotic fluctuations (Supplementary Fig. 4). Overall, the function and evolution of methanogenic ecosystems lead to a less resilient, more variable climate.

The formation of organic hazes when the *p*CH_4_-to-*p*CO_2_ ratio is greater than 0.2 (ref. ^24^) has been proposed as a general mechanism of climate regulation^25^. In the late Archean, *p*CO_2_ was low^5^, favoring organic haze formation that may have prevented hot runaway scenarios. In the Hadean/early Archean, however, our model predicts organic hazes to form in a limited range of conditions, at very high H_2_ volcanic outgassing rates, low *p*CO_2_, and low abiotic temperature close to or below the freezing point (Fig. 3). Under these specific conditions, the formation of organic hazes may overwhelm the warming effect of methanogenic ecosystems and leave the planet in a globally glaciated state. Under most conditions, however, it is the availability of electron donors (H_2_, CO) to methanogenesis, and not organic hazes, that is expected to limit Archean climate warming by biological activity.

### Biomass production

In spite of their strong impact on the planet’s atmosphere and climate, primitive methanogenic ecosystems are characterized by extremely low biomass productivity, AG + AT being the most productive pathway by far (Fig. 2). As microbial chemolithotrophs consume atmospheric electron donors, they drive the system closer to its thermodynamic equilibrium, thus gradually decreasing the thermodynamic efficiency of the metabolic coupling between energy acquisition (catabolism) and biomass production (anabolism; see “Methods equation (E2)”). As a consequence, for the highest value of abiotic H_2_ outgassing, our model predicts biomass production to range from 10^6^ to 10^9^ molecule C cm^−2^ s^−1^. This is 1–4 orders of magnitude below previous estimates^13^ (based on models that assumed a fixed biomass yield per electron donor consumed) and 4–7 orders of magnitude below modern values^26^.

Albeit extremely low, biomass production is very sensitive to the metabolic composition of the ecosystem and temperature, *T*_Geo_. Supplementary Fig. 5 shows how biomass production is influenced by these two factors. For the MG ecosystem, biomass production peaks for *T*_Geo_ between −10 and 10 °C depending on the intensity of H_2_ volcanic outgassing (slightly above 10^9^ molecules C cm^−2^ s^−1^ at high rates of H_2_ volcanic outgassing), and strongly decreases for higher *T*_Geo_. The maximum biomass production is of the same order in the AG + AT ecosystem and reached for similar conditions (intermediate *T*_Geo_, high H_2_ volcanic outgassing rate), but its dependence upon *T*_Geo_ and the rate of H_2_ volcanic outgassing is much weaker. In the MG + AG + AT ecosystem the two methanogenic pathways interact synergistically, leading to a nonlinear, multiplicative increase in biomass production at low and high temperature. The synergy involves the combination of biogeochemical recycling loops, both locally and globally. Locally, while MG consumes CO_2_ to produce CH_4_, AT decomposes CH_3_COOH and produces CO_2_. The metabolic waste of AT is, therefore, the metabolic substrate of MG, and the combination of the two metabolic pathways pulls the system further away from its thermodynamic equilibrium, hence an increase in the efficiency of both pathways. Globally, as the MG metabolism releases additional CH_4_ in the atmosphere, the production of CO through photochemistry is accelerated; CO being the metabolic substrate of AG, its metabolic efficiency is enhanced. The synergistic effect is greatest at low abiotic temperature. In those very specific conditions and for the highest values of H_2_ outgassing, biomass production can reach about 10^10^ molecules C cm^−2^ s^−1^—about 1000 times less than estimates of modern primary production^26^.

### Metabolic evolution and the carbon cycle

**Fig. 4:**
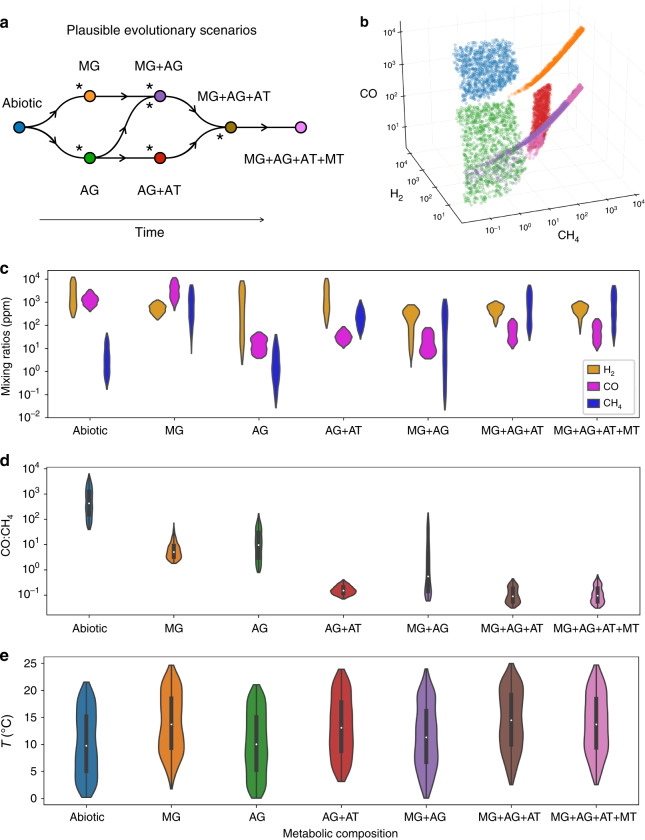
Equilibrium state of the planet as the biosphere diversifies. **a** Plausible evolutionary sequences of metabolic innovation. Asterisks denote transitions that are very likely to cause a significant change in the atmospheric composition. **b** Scatterplot of the atmospheric compositions at equilibrium in CO, CH_4_, and H_2_, color-coded by the corresponding biosphere composition (1000 simulations for each ecosystem). **c** Corresponding distributions. **d** Distribution of the CO:CH_4_ ratio for each scenario. **e** Distribution of the surface temperature in each scenario. The white dots in **d**, **e** represent the median of the distributions, the thick gray lines the interquartile range, and thin gray lines the rest of the distribution.

Next, we investigate how the evolutionary process of metabolic diversification of the primitive biosphere shaped the planet atmospheric and climatic history. We consider alternate scenarios of biosphere evolutionary complexification (Fig. 4a) consistent with phylogenetic and geological inferences^2,7,9–11,27^. Our evolutionary sequences culminate with the evolution of anaerobic methanotrophy, which uses the oxidation of CH_4_ as primary source of energy, based on the consumption of H_2_SO_4_, the main oxidative species of the globally reduced early Earth^28^. We, therefore, make the plausible assumption that a full methane-cycling biosphere may have evolved before the advent of photosynthesis.

This evolutionary process of diversification may have spanned several hundred million years (from the origin of life 3.9–4.5 Gya, to the origin of anoxygenic phototrophy, 3.5–3.7 Gya), thus unfolding on a timescale over which the geochemical cycles interacted dynamically and reciprocally with biological activity. In particular, it has been shown^5^ that on the timescale of 10^7^ to 10^8^ years, the carbon cycle tends to mitigate temperature variations through a negative feedback on the atmospheric CO_2_ concentration. We adapted the model from ref. ^5^ to add a full, temperature-dependent carbon cycle to our planetary ecosystem model. The abiotic equilibrium *p*CO_2_ is now determined by the balance between CO_2_ outgassing and sequestration in the oceanic floor; *p*CH4, by the balance between serpentinization and photodissociation rates; and *p*H_2_, by the balance between outgassing and photochemical reactions. Additionally, the H_2_SO_4_ oceanic concentration is determined by the balance between rainout and hydrothermal remineralization rates (taken from ref. ^29^). By drawing values for the abiotic supplies in CO_2_, CH_4_, H_2_, and H_2_SO_4_ from reasonable, log-uniform priors (Table 1), and starting from a lifeless primitive Earth, we evaluate the equilibrium state of the planetary system after each evolutionary metabolic transition in terms of atmospheric signatures (Fig. 4b–d) and mean surface temperature (Fig. 4e).

**Table 1:** Abiotic inputs of CO_2_, H_2_, CH_4_ and H_2_SO_4_.

Abiotic inputs	Range (in molecules cm^−2^ s^−1^)	Reference
Volcanic output of CO_2_	1.2 × 10^10^– 4 × 10^10^ (to obtain *T*_Geo_ ranging from 0 to 20 °C)	ref. ^5^
Volcanic output of H_2_	5 × 10^9^–3 × 10^11^	ref. ^12^
Serpentinization rate of production of CH_4_	3.7 × 10^8^–3.7 × 10^9^	ref. ^33,42^
Deposition rate of H_2_SO_4_	10^7^–10^9^	ref. ^30^

First, we find that a lifeless Earth is characterized by a high CO:CH_4_ atmospheric ratio of 10^2^–10^4^ to 1, which differs markedly from the ratio predicted with a functional biosphere, regardless of its metabolic composition. As the biosphere complexifies, biological activity increases the atmospheric concentration in CH_4_, decreases the atmospheric CO, or both, causing the CO:CH_4_ ratio to fall. By comparing median values between the abiotic state and the most complex biosphere (MG + AG + AT + MT), the CO:CH_4_ ratio is predicted to be reduced by a factor of ~5000. The earliest evolutionary events, whereby the MG, AG, or AG + AT ecosystem emerges, all cause atmospheric shifts that can be distinguished in the *p*CO-*p*H_2_-*p*CH_4_ space (Fig. 4b and c). The atmospheric shifts caused by subsequent evolutionary complexification (leading to MG + AG, MG + AG + AT, or MG + AG + AT + MT ecosystems) are less pronounced and the corresponding atmospheric signatures are less distinctive among themselves. The evolution of anaerobic methanotrophy (MT) has for instance no effect on the equilibrium atmosphere of the planet because the influx of H_2_SO_4_ is sufficient for methanotrophs to survive and co-occur with methanogens, but not for them to consume a significant portion of the CH_4_ produced by methanogens.

Finally, although methanogenesis can have major effects on climate on relatively short time scales (Figs. 2 and 3), the carbon cycle buffers these effects at equilibrium on longer time scales: the average temperature difference between the planet with and without a methanogenic biosphere on its surface is only 4 °C (Fig. 4d). Biological effects on temperature may be further attenuated by the formation of cooling organic hazes favored by the enhanced sequestration of CO_2_ in response to methanogenesis^25^. However, the necessary conditions for organic hazes to form in this case are met in only 0.03% of the simulations including a methanogenic biosphere.

### Transient climate destabilization by methanotrophy

How the planetary atmosphere-climate system responds to metabolic innovation may depend on the pace of evolution itself. The evolution of methanotrophy is a case in point. Slow evolution may delay methanotrophy after the response of the carbon cycle to methanogenesis. In this case, the oceanic stocks of H_2_SO_4_ are sufficient for methanotrophs to rapidly consume almost all of the atmospheric CH_4_ (Supplementary Fig. 6) and the planet is temporarily characterized by an atmospheric deficit in both CO_2_ and CH_4_. As a result, temperature plummets below the initial abiotic temperature. In contrast, if evolution is fast enough and methanotrophs evolve before equilibration of the methanogenic biosphere with the planetary atmosphere and climate, atmospheric CH_4_ may be consumed during or after the warming period (Fig. 3) but before the deficit in *p*CO_2_ builds up; temperature then returns close to its initial value (Supplementary Fig. 6).

**Fig. 5:**
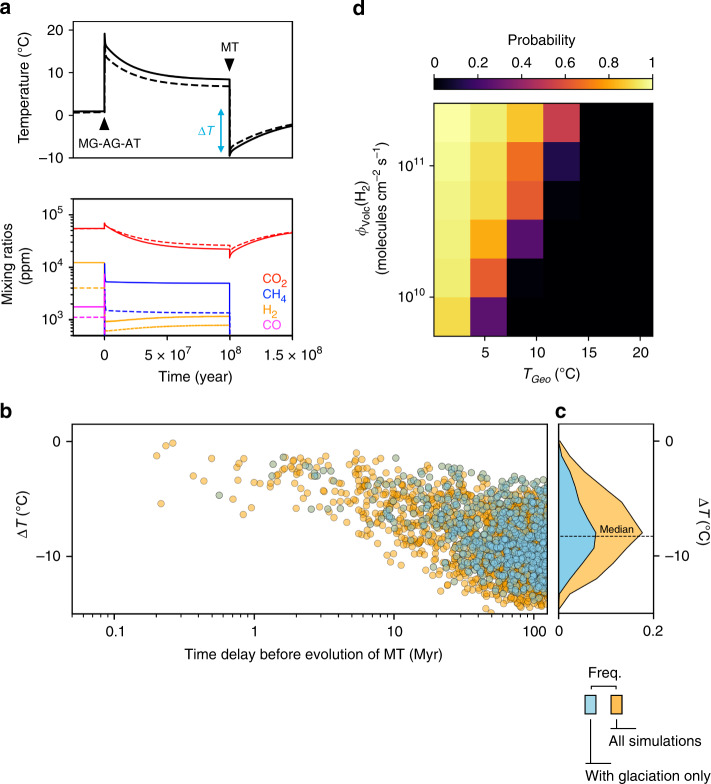
Climate destabilization by evolutionary metabolic innovation. **a** In this example, sulfur-based methanotrophy (MT) evolves 100 million years after MG + AG + AT, i.e. after equilibration of the methanogenic biosphere (MG + AG +AT) with the atmosphere and climate mediated by the carbon cycle, with *T*_Geo_ = 2 °C, *φ*_volc_(H_2_) = 3 × 10^11^ (plain lines) and 1 × 10^11^ molecules  s^−1^ cm^−2^ (dotted lines). Top, change in surface temperature. Bottom, change in atmospheric composition. **b**, **d** Distribution of outcomes across a range of abiotic temperature *T*_Geo_, H_2_ volcanic flux, and evolution time of MT (2000 randomly chosen combinations). **b** Amplitude of global cooling, ∆*T*, with respect to the evolution time of MT. **c** Frequency distribution of all temperature changes ∆*T* (blue) and of temperature changes conditional on glaciation outcome (yellow). **d** Estimated probability of glaciation as a consequence of MT evolution, given the abiotic temperature *T*_Geo_ and H_2_ volcanic flux. Other parameters are set to their default values (Supplementary Tables 2 and 3).

Figure 5a illustrates the atmospheric and climatic consequences of slow evolution, when the wait time for methanotrophy is of the order of the carbon cycle timescale. In the two examples shown, the evolution of methanotrophs causes the sharp climatic response described above, and temperature falls respectively by 8 and 10 °C below *T*_Geo_ = 2 °C, driving the planet into a global glaciation. Among 2000 simulated planetary conditions characterized by randomly drawn abiotic characteristics (i.e., the abiotic influxes of CO_2_, H_2_, CH_4_ and H_2_SO_4_, thereby setting the abiotic *p*CO_2_, *p*H_2_, *p*CH_4_, initial oceanic concentration of H_2_SO_4_ and surface temperature *T*_Geo_), and evolution time of methanotrophy (from 0 to 125 million years after methanogenesis), 50% experience a temperature drop larger than 8.3 °C below *T*_Geo_ (Fig. 5b), and 40% actually end up in glaciation (Fig. 5b and Supplementary Fig. 7). As expected, delayed evolution of methanotrophy makes extreme cooling more likely (Fig. 5b). Because they lead to an enhanced abiotic response of the carbon cycle, conditions for which methanogenic ecosystems have the greatest warming effect (low abiotic *T*_Geo_, high H_2_ outgassing rate) are also the conditions under which the evolution of methanotrophy has the most dramatic effect on climate and habitability (Fig. 5a and c, Supplementary Fig. 8). Noticingly, this transient occurs irrespective of the metabolic composition of the methanogenic biosphere prior to the evolution of methanotrophy, as illustrated in Supplementary Fig. 7.

For a methane-cycling ecosystem that triggers and eventually survives global glaciation, we expect the resulting equilibrium (co-occuring methanogens and methanotrophs with low *p*CH_4_) to be maintained only transitorily. We assume that during the early Archean, prior to the evolution of methanotrophy, the oceanic stock of H_2_SO_4_ builds up as volcanoes emit SO_2_ photochemically transformed into H_2_SO_4_, which then deposits on the ocean surface. With a deposition rate corresponding to our lowest value of 10^7^ molecules cm^−2^ s^−1^ (ref. ^30^), and a hydrothermal removal rate of [H_2_SO_4_]_oc._ × 7.2 × 10^12^ L y^−1^ (ref. ^29^), we obtain an abiotic oceanic concentration of 0.4 mM. Such a stock is sufficient for methanotrophs to consume most of the atmospheric CH_4_, leading to the global cooling described above. Since the rate of CH_4_ reduction by methanotrophs is faster than the deposition rate, the H_2_SO_4_ stock will ultimately be depleted by methanotrophs, thereby driving a new abrupt environmental shift towards the equilibrium described in Fig. 4. At this new, stable equilibrium of coexisting methanogens and methanotrophs, the atmospheric *p*CH_4_ is high and the H_2_SO_4_ oceanic concentration is very low, below the micromolar, which aligns with the sulfur isotopic fractionation records^30–32^. This result suggests that anaerobic methanotrophy may have been the main sink of H_2_SO_4_ prior to the Neoarchean (2.5–2.7 Gya).

## Discussion

Our initial questions were: How constrained was the habitability of late Hadean/early Archean Earth to methane-cycling ecosystems? How productive were these ecosystems and did they have a significant impact on the atmospheric composition and climate? How did their impact change as different metabolisms evolved? To address these questions, we build on the previous theory^8,12,13^ by setting up a probabilistic modeling framework in which the evolution of a microbial biosphere is coupled to the dynamics of early Earth surface geochemistry and climate. We focus on four simple microbial metabolisms involved in methane cycling and likely to be some of the earliest players in Earth’s ecology: hydrogenotrophic methanogenesis (MG), acetogenesis (AG), methanogenic acetotrophy (AT), and anaerobic oxidation of methane (MT). The biosphere evolves when a metabolism that was not present appears and adapts. By closing the global feedback loop between biological and planetary surface processes, the model predicts both ecosystem and atmosphere-climate states under the assumption that they reciprocally influence one another.

Our results confirm the contention that the late Hadean/early Archean planet was most likely habitable to methane-cycling chemolithotrophic biospheres and that under the assumption of high enough H_2_ supply, these biospheres were key factors of the climatic and atmospheric evolution of the planet^8,12,13,33,34^. On short time scales (10^5^–10^6^ years) the evolution of methanogenic biospheres may have considerably warmed the climate and influenced its resilience, in spite of a very low ecosystem productivity. On longer timescales, commensurate with the abiotic response of the carbon cycle to temperature variation, all ecosystems converge to new stable equilibria. Under these long-term equilibrium conditions, the mean surface temperature does not differ much from the lifeless state, and the carbon cycle is the predominant mechanism of climate regulation. However, all ecosystem equilibria share a robust atmospheric signature (low CO:CH_4_ ratio), distinctive from the lifeless state. In addition to influencing planetary characteristics at equilibrium, metabolic evolution generates atmospheric and climatic transients. The pace of evolution thus has a strong influence on the atmosphere and climate history. In particular, fast evolution of methanotrophs has limited or no effect on climate, whereas their delayed evolution may cause strong transients leading to global glaciation.

Although the influence of chemotrophic methane-cycling ecosystems on climate has been discussed in the previous work^8,12,13,33,34^, our model is the first to couple models of the Archean atmosphere, climate, and carbon cycle to an explicit eco-evolutionary model of cell population dynamics in order to quantify this effect. Our model differs from previous work primarily by addressing how climate change driven by ecological function feeds back to the biological activity of microbial populations, through the thermal dependence of the thermodynamics and kinetics of cell metabolism, and triggers an abiotic response of the carbon cycle. Closing the global feedback loop between ecological and planetary processes allows us to predict the ecosystem and climate states under the assumption that they reciprocally influence one another on multiple timescales.

A general result is that MG and AG + AT ecosystems are characterized by extremely low biomass production relative to their planetary impact on the atmosphere and climate. We predict biomass production to be 1–4 orders of magnitude smaller than previous estimates^13^ and 3–7 orders of magnitude below modern values^26^. Maximum global biomass production (reached for a very specific combination of biotic and abiotic conditions) is 10^10^ molecules C cm^−2^ s^−1^, or 3 × 10^7^ ton C year^−1^. This is ~1000 times less than estimates of modern primary production. Both in terms of stock and fluxes, biomass has therefore very little effect on the biogeological coupling, which is a major difference with modern ecosystems.

A key assumption of our model is that nutrients N and P are not limiting; this assumption is backed up by the prediction of very low biomass production. As previously argued^13,14^, primitive ecosystems with such low productivity should have been limited by the availability in electron donors rather than by N and P nutrients. More specifically, ref. ^14^ evaluated the most likely limitation to biomass production prior to the evolution of oxygenic photosynthesis. By assuming a fixed yield for biomass production per molecules of electron donor consumed (as in ref. ^13^), they found that the N and P supplies were most likely sufficient during the Archean for electron donors to be limiting. This is even more likely given our finding of a biomass productivity far lower than the previous estimates^13,14^.

Our prediction of very low biomass productivity may help better constrain the timeline of the evolution of metabolic innovation on Earth. Even in our most productive scenario and under the (extreme) assumption that 100% of the dead organic matter is buried before remineralization, the estimated biomass production is still 4–5 times lower than the level consistent with the C isotopic fractionation estimated from rocks as old as 3.5 Gy^1,35^. This suggests that more productive, likely photosynthetic life forms must have evolved more than 3.5 Gya ago.

By performing equilibrium analyses of the planetary system on longer time-scales (10^7^–10^8^ years), on which the carbon cycle responds to ecosystem function and sequential metabolic diversification of the biosphere, we find that the climate regulation of the planet by the abiotic carbon cycle largely buffers the influence of early methanogenic activity on climate. However, depending on the pace of evolution, metabolic transitions such as the evolution of methanotrophy can interact with the abiotic carbon cycle and trigger strong transitory climatic events such as global glaciation over 10^7^–10^8^ years.

Even though the timing of the origin of anaerobic oxidation of methane (AOM), by reduction of nitrate, nitrite, or sulfate, is not well resolved, the fact that AOM proceeds enzymatically as a reversal of methanogenesis has been used to suggest that AOM may have evolved relatively soon after methanogens, i.e. before phototrophy^36,37^. Additionally, we find that the evolution of AOM prior to photosynthesis appears to be compatible with the sulfur isotopic fractionation record^30–32^. Our results thus highlight biological activity and the evolution of pre-photosynthetic methane-cycling ecosystems as a potential destabilizing factor of the early Earth climate system, alternatively or additionally to abiotic causes such as large impactors^3,4,38^.

The mechanism by which the evolution of early methane-cycling ecosystems may have exposed the Earth to high risks of global glaciation—fast removal of atmospheric methane, slow response of the carbon cycle—is general. It is, therefore, tantalizing to speculate about its potential involvement in the major climatic events that paralleled the evolution of oxygenic photosynthesis. By poisoning methanogens, removing the resource limitation (oxidizing species: O_2_, H_2_SO_4_, NO_3_, NO_2_) of methanotrophs, or by simply driving the abiotic oxidation of atmospheric CH_4_ by outgassed O_2_, oxygenic photosynthesis may have driven a dramatic decline in atmospheric CH_4_ which, coupled to a delayed response of the carbon cycle, could have caused global cooling, triggering the Proterozoic glaciation ~2.3 Gya^39–41^.

The substantial shifts in atmospheric composition driven by evolutionary metabolic innovation (Fig. 4) highlight the fact that simple methane-cycling ecosystems can have radically different atmospheric signatures. Thus, the atmosphere composition is shaped very early on by the course of biological evolution. We observe that the atmospheric signature of a specific metabolism is highly dependent on the ecological context set by the whole metabolic composition of the biosphere. As the biosphere evolves and complexifies, the atmospheric signature of each metabolic type becomes less identifiable, yet the state of the planet remains distinctive from the lifeless scenario. This backs up and quantifies the hypothesis that on a globally reduced planet such as the early Archean Earth, a low CO:CH_4_ atmospheric ratio is a highly discriminant indicator of inhabitation by a primitive CH_4_-based biosphere^42^.

In conclusion, our results suggest that life has had a dramatic impact on the anoxic Earth’s surface environment, long before phototrophy evolved. They highlight the importance of the evolutionary process and its timeline in shaping up the planet’s atmosphere and climate. In spite of extremely low productivity, metabolic evolutionary innovation in primitive methane-based biospheres is predicted to cause distinctive shifts in atmospheric composition, such as a decreasing CO:CH_4_ ratio as greater metabolic complexity evolves. The warming effect of methanogens and cooling effect of methanotrophs can be strong but they are only transient, on timescales that depend on the pace of evolution. We anticipate that the continued development of models that couple planetary processes with ecological and evolutionary dynamics of microbial biospheres will further advance our understanding of major events in the co-evolutionary history of life and Earth, and help identify detectable biosignatures for the search of life on Earth-like exoplanets.

## Methods

### Biological model

In the following section, we present the biological model. Parameters’ definition, unit and default value are given in Supplementary Tables 1, 2, and 3.

The biological model describes the dynamics of one or several biological populations of chemotrophic organisms, driven by the growth, birth (division) and death of individual cells. The individual cell life cycle is controlled by catabolic and anabolic reactions occurring within cells^17,18,43^. Catabolism produces energy used by anabolism for biomass production, which determines cell growth and division; a fraction of energy produced by catabolism is used for cell maintenance. Energy thus flows from catabolism to maintenance and anabolism, and these processes can be described as follows:

E1 $$\begin{array}{*{20}{l}} {{\mathrm{Catabolism}}} \hfill & : \hfill & {\mathop {\sum }\limits_{i = 1}^n \gamma _{S_i}^{cat}S_i \to \mathop {\sum }\limits_{i = 1}^m \gamma _{P_i}^{cat}P_i + {\mathrm{\Delta }}G_{cat}} \hfill \\ {{\mathrm{Maintenance}}} \hfill & : \hfill & {E_m} \hfill \\ {{\mathrm{Anabolism}}} \hfill & : \hfill & {\mathop {\sum }\limits_{i = 1}^{\dot n} \gamma _{S_i}^{cat}S_i + {\mathrm{\Delta }}G_{ana} \to \mathop {\sum }\limits_{i = 1}^{\dot m} \gamma _{P_i}^{cat}P_i + B} \hfill \end{array}$$

where *S*_*i*_ and *P*_*i*_ are the substrates and products of the metabolic reactions, and the $\gamma$’s are the corresponding stoichiometric coefficients. We follow ref. ^17^ and assume that the average organic compound ${\mathrm{C}}{\mathrm{H}}_{1.8}{\mathrm{O}}_{0.5}{\mathrm{N}}_{0.2}$ is a plausible approximation for the living biomass *B*. The energy *E*_*m*_ (in kJ d^−1^) measures the cost of maintenance for a single cell per unit of time. The ∆*G* terms measure the oxidative power released or consumed by the catabolic and anabolic reactions; they are given by the Nernst relationship:

E2 $${\mathrm{\Delta }}G(T) = {\mathrm{\Delta }}G_0(T) + {RTlog} \left(\mathop {\prod }\limits_{i = 1}^n S_i^{ \gamma _{S_i}}\mathop {\prod }\limits_{i = 1}^m P_i^{\gamma _{P_i}}\right)$$

where *R* is the ideal gas constant, *T* is the temperature (in K) and ∆*G*_0_(*T*) (in kJ) is the Gibbs energy of the reaction. ∆*G*_0_(*T*) is obtained from the Gibbs–Helmholtz relationship:

E3 $${\mathrm{\Delta }}G(T) = {\mathrm{\Delta }}G_0(T_S)\frac{T}{{T_S}} + {\mathrm{\Delta }}H_0(T_S)\frac{{T_S - T}}{{T_S}}$$

where *T*_*S*_ is the standard temperature of 298.15 K. Note that equations (E2) and (E3) describes how the thermodynamics of any given metabolism (i.e., the combination of catabolism and anabolism) vary with temperature.

Michaelis–Menten kinetics apply to catabolism:

E4 $$q_{cat} = q_{max}\frac{{min(S_i^{cat}/\gamma _{S_i}^{cat})}}{{min(S_i^{cat}/\gamma _{S_i}^{cat}) + K_S}}$$

where *K*_*S*_ is the half-saturation constant, *q*_*max*_ the maximum metabolic rate of the cell (*d*^−1^), and $min(S_i^{cat}/\gamma _{S_i}^{cat})$ measures the concentration of the limiting substrate (taking stoichiometry into account). The energy produced is first directed toward maintenance. The cell energetic requirement for maintenance is:

E5 $$q_m = \frac{{ - E_m}}{{{\mathrm{\Delta }}G_{cat}}}$$

The cell therefore meets its energy requirement only if *q*_*cat*_ > *q*_*m*_. If the cell does not meet this requirement, the basal mortality rate of the cell, *m*, (in *d*^−1^), is augmented by a decay-related mortality term equal to $k_d(q_m - q_{cat})$. Hence the actual mortality rate:

E6 $$\begin{array}{*{20}{l}} {If\,q_{cat} \,<\, q_m} \hfill & : \hfill & {d = m + k_d(q_m - q_{cat})} \hfill \\ {If\,q_{cat} \,> \, q_m} \hfill & : \hfill & {d = m} \hfill \end{array}$$

When the energy requirements for maintenance are not met (*q*_*cat*_ < *q*_*m*_) no energy is allocated to biomass production. Conversely, when those requirements are met (*q*_*cat*_ < *q*_*m*_), then the energy remaining after allocation to cell maintenance can be directed to anabolism. A constant quantity of energy${\mathrm{\Delta }}G_{diss}$ (in kJ) is then lost through dissipation. Following ref. ^17^ we consider the following empirical relationship:

E7 $${\mathrm{\Delta }}G_{diss} = 200 + 18(6 - NoC)^{1.8} + e^{( - 0.2 - \alpha )^{2.16}(3.6 + 0.4NoC)}$$

where *NoC* is the number of carbons in the carbon source used by the anabolic reaction, and $\alpha$ is its degree of oxidation. The efficiency of metabolic coupling (i.e., the number of occurrences of the catabolic reaction to fuel one occurrence of the anabolic reaction once the maintenance cost has been met) is then measured by

E $$\lambda = \frac{{ - {\mathrm{\Delta }}G_{cat}}}{{{\mathrm{\Delta }}G_{ana} + {\mathrm{\Delta }}G_{diss}}}$$

The Michaelis–Menten kinetics of anabolism is given by

E9 $$\begin{array}{*{20}{l}} {If\,q_{cat} \,> \, q_m} \hfill & : \hfill & {q_{ana} = \lambda (q_{cat} - q_{m})\frac{{min(S_i^{ana}/\gamma _{S_i}^{ana})}}{{min(S_i^{ana}/\gamma _{S_i}^{ana}) + K_S}}} \hfill \\ {If\,q_{cat} \,<\, q_m} \hfill & : \hfill & {q_{ana} = 0} \hfill \end{array}$$

where the half-saturation constant *K*_*S*_ independent of the substrate, as was assumed in ref. ^43^. As biomass accumulates in the cell at rate *q*_*ana*_, cell growth may lead to cell division. The cell division rate, *r*, is given by:

E10 $$\begin{array}{*{20}{l}} {If\,B \,<\, 2B_{Struct}} \hfill & : \hfill & {r = 0} \hfill \\ {If\,B \,> \, 2B_{Struct}} \hfill & : \hfill & {r = r_{max}\frac{1}{{1 + e^{ - \theta log10((B - 2B_{Struct})/B_{Struct})}}}} \hfill \end{array}$$

This means that a cell cannot divide if its internal biomass is not at least twice its cell structural biomass so that the two daughter cells meet this structural requirement. Above this threshold value of 2*B*_*Struct*_, *r* is of sigmoidal form so that the division rate first increases exponentially with the intracellular biomass content then saturates to *r*_*max*_ when biomass is largely available.

Using the rates of catabolic and anabolic cell activity and resulting cell division and mortality rates, we derive the following system of ordinary differential equations driving the cell population dynamics (dynamics of the number of cells and average cell biomass) and the feedback of the population on its environment (chemical composition of the ocean surface):

E11 $$\begin{array}{*{20}{l}} {\frac{{dN_i}}{{dt}}} \hfill & = \hfill & {(r_i - d_i)N_i} \hfill \\ {\frac{{dB_i}}{{dt}}} \hfill & = \hfill & {q_{ana_i} - r_iB} \hfill \\ {\frac{{dX_j}}{{dt}}} \hfill & = \hfill & {F(X_j) + \mathop {\sum }\limits_{i = 1}^{MT} (q_{cat_i}\gamma _{i,X_j}^{cat} + q_{ana_i}\gamma _{i,X_j}^{ana})N_i} \hfill \end{array}$$

where *MT* denotes the ensemble of metabolic types considered, *N*_*i*_ is the number of individual cells in a population of a given metabolic type, *B*_*i*_ is the average cellular biomass of that type, and $X_1,...,X_S$ are the concentrations of all relevant chemical species in the environment. The term ${\sum }_{i = 1}^{MT} (q_{cat_i}\,\gamma _{i,X_j}^{cat} + q_{ana_i}\gamma _{i,X_j}^{ana})N_i$ describes how concentrations vary according to the biological activity of each biological population *i* in the microbial community. The *F*(*X*_*j*_) terms describe the environmental forcing resulting from ocean circulation and atmosphere-ocean exchanges as simulated by a stagnant boundary layer model as in ref. ^13^. The system of equations (E11) is solved numerically using a forward Euler method.

The flow of energy through the cell is driven by the maximum metabolic rate, *q*_*max*_ (in *d*^−1^), and the rate of energy consumption for maintenance, *E*_*m*_ (in kJ d^−1^). They are both expressed as functions of temperature and cell size of the form *e*^*a*+*b*^*T*V^c^. Default values for parameters *a*, *b* and *c* entering *q*_*max*_ and *E*_*m*_ are given in Supplementary Table 2.

The structural cell biomass *B*_*Struct*_ increases with cell size according to *B*_*Struct*_  = *aV*^*b*^. Both metabolic rate and maintenance cost increase with cell size, but not as fast as structural biomass. Consequently, the biomass specific rates of metabolism and energy consumption for maintenance decrease with cell size. Thus, small organisms are better at acquiring energy, but large organisms are more cost efficient due to lower maintenance requirements. A trade-off mediated by cell size thus exists between metabolic and maintenance rates, hence an optimal (intermediate) cell size, which is consistent with previous work conducted on unicellular marine organisms^22^. Both metabolic and maintenance rates increase with temperature, but the metabolic rate increases slightly faster^19,20^, shifting the trade-off toward larger sizes. As a consequence, the optimal cell size increases with temperature.

To compute the evolutionarily optimal cell size for a given metabolic type at a given temperature, we run simulations across a range of cell size and measure the level of resource use at equilibrium given by $Q^ \ast = \mathop{\prod}\limits_{i = 1}^n {S_i^{ \gamma _{S_i}^{cat}}} \mathop{\prod}\limits_{i = 1}^m {P_i^{\gamma _{P_i}^{cat}}}$, the product of the metabolic substrates and wastes concentrations at equilibrium weighted by their stoichiometric coefficient. According to the classical principle of “pessimization” (maximization of resource use)^44–46^, populations that are better at exploiting their environment have lower Q^*^ and evolution by natural selection will favor the cell size that minimizes Q^*^, thus leading to the evolutionarily optimal cell size. Supplementary Fig. 9 shows Q* as a function of cell size and temperature for the H_2_-based methanogenesis.

The optimal cell size, *S*_C_^*^, follows a positive relationship with temperature given by *S*_C_^*^ = 10^*a*+*bT*^. We consider that over geological time scales, adaptive evolution acting on genetic variation among cells is fast enough so that *S*_*C*_ is equal to *S*_C_^*^. As temperature changes, evolutionary adaptation by natural selection tracks the temperature-dependent optimal cell size.

In contemporary Earth oceans, most of the dead biomass is recycled by fermentors (>99%), and the rest is buried into the ocean floor. Although the most general version of our model includes populations of acetogenic biomass fermentors and acetotrophs, their inclusion considerably increases simulation time (results not shown). All the results presented in the main text have been obtained without fermentors, assuming that the dead biomass accumulates in the ocean’s interior. It appears that biomass productivity is so low that the effect of biomass recycling (or lack thereof) on the atmospheric composition is negligible. We verified this by testing the effect of a recycling pathway in each ecosystem (MG, AG + AT, MG + AG + AT), for an intermediate value of H_2_ volcanic outgassing. Although biomass production is sufficient to sustain a population of fermentors, we found no significant effect of their biological activity on the atmospheric composition. Additionally, when we can compare the global atmospheric redox budget of the planet with and without biomass production (see Supplementary Results and Discussion, Supplementary Fig. 12), we find no significant differences. This further demonstrates that biomass production and the accumulation of dead biomass in the absence of remineralization represents a sink of C and H that is marginal compared to the other fluxes in the model. Note that the situation would be very different after the evolution of more productive, photosynthetic primary producers.

Because we do not model the fate of dead biomass explicitly, we evaluate the consistency of our predictions of biomass production with the geological record (carbon isotopic fractionation data) by taking biomass production as the upper bound for burial (in which case 100% of the produced biomass is ultimately buried) and using the modern value of burial (taken to be 0.2% as in e.g., ref. ^13^) as lower bound.

### Planetary model

Our computation of climate state and mean surface temperature is based on 3D simulations with the Generic LMD GCM^4,47,48^. The model includes a 2-layer dynamic ocean computing heat transport and sea ice formation^49^. The radiative transfer is based on the correlated-*k* methods with *k*-coefficients calculated using the HITRAN 2008 molecular database. We used the simulations described in ref. ^4^ for the early Earth at 3.8 Ga, assuming no land, 1 bar of N_2_ and a 14 h rotation period. The simulation grid covers a range of *p*CO_2_ from 0.01 to 1 bar and pCH_4_ from 0 to 10 mbar. From this grid, we derived the following simple parametrization of the mean surface temperature as a function of *p*CO_2_ and *p*CH_4_ (expressed in bar):

E12 $$T\left( {\! \deg {\mathrm{C}}} \right) = - 19.26 + 77.67 {\sqrt {p{\mathrm{C}}{{\mathrm{O}}_2}}} \,\,+\, 5{\mathrm{log}}10\left( {\frac{{1 + p{\mathrm{C}}{{\mathrm{H}}_4}{{10}^6}}}{3}} \right)$$

In the coupled biological-planetary model, we assume that the climate is always at equilibrium, meaning that the timescale of climate convergence is shorter than biological and geochemical timescales.

Photochemistry is computed with the 1D version of the Generic LMD GCM, which now includes a photochemistry core from ref. ^50^. The chemical network includes 30 species (mostly hydrocarbons) and 114 reactions. We use the reactions from ref. ^50^ for H_2_-H_2_O-CO_2_ and from ref. ^51^ for hydrocarbons. The photochemistry model also includes a pathway for the formation of hydrocarbon aerosol (C_2_H + C_2_H_2_ → HCAER + H)^51,52^.

We use the eddy diffusion vertical profile from ref. ^53^ and the solar UV spectrum at 3.8 Ga from ref. ^54^. Boundary conditions are set by the mixing ratios of CO_2_, CH_4_, and H_2_O at the first atmospheric layer. The atmospheric CO is either consumed biotically by acetogens when they are present in the biosphere, or abiotically by the formation of formate in the ocean (resulting in a deposition velocity of ~10^8^ cm s^−1^) as in ref. ^13,55^. This eliminates the possibility of CO-runaway, which otherwise occurs in our simulations at high level of H_2_ or CO_2_. In addition, we assume that H_2_ undergoes diffusion-limited atmospheric escape (see for instance ref. ^13^).

We performed simulations across a range of *p*CO_2_ (10^4^–10^5^ ppm), *p*CH_4_ (1–10^4^ ppm), H_2_ (100–10^4^ ppm). For our range of atmospheric composition, photochemistry is dominated by the photolysis of CO_2_ and CH_4_, whose net reactions are:

R1 $${\mathrm{CO}}_2 + {\mathrm{H}}_2 = {\mathrm{CO}} + {\mathrm{H}}_2{\mathrm{O}}$$

R2 $${\mathrm{CH}}_4 + {\mathrm{CO}}_2 = 2{\mathrm{CO}} + 2{\mathrm{H}}_2$$

From the simulation outputs we derived simple parametrizations for the production rates and loss rates of CH_4_, CO, H_2_ (see Supplementary Fig. 10). The reaction rates of (R1) and (R2) can be parameterized respectively as (in molecules cm^−2^ s^−1^):

E13 $$F_1 = 1.8\,10^{10} \cdot \left( {\frac{{p{\mathrm{C}}{\mathrm{O}}_2}}{{10^{ - 1}}}} \right) \cdot \left( {\frac{{p{\mathrm{H}}_2}}{{10^{ - 4}}}} \right)^{0.4}$$

E14 $$F_2 = 2\,10^{10}\left( {\frac{{p{\mathrm{C}}{\mathrm{O}}_2}}{{10^{ - 1}}}} \right)^{ - 0.2} \,\cdot\, \left( {\frac{{p{\mathrm{H}}_2}}{{10^{ - 4}}}} \right)^{ - 0.2} \,\cdot\, \left( {\frac{{p{\mathrm{C}}{\mathrm{H}}_4}}{{10^{ - 4}}}} \right)^u$$

where *u* (between 0.5 and 1) is given by

E15 $$u = 0.6 + 0.1{\it{{\mathrm{log}}}}\left( {\frac{{p{\mathrm{H}}_2}}{{10^{ - 4}}}} \right) - \frac{{{\it{{\mathrm{log}}}}({\it{p{\mathrm{C}}{\mathrm{H}}}}_4) + 2}}{{15}} + 0.08\log \left(\frac{{p{\mathrm{C}}{\mathrm{O}}_2}}{{10^{ - 1}}}\right)$$

To simulate the evolution of *p*CO_2_ with time and feedbacks between the microbial community, climate, and the carbon cycle, we use a simple carbon cycle model based on ref. ^5^. The model computes the evolution of atmospheric CO_2_, dissolved inorganic carbon (CO_2_, HCO_3_^−^ and CO_3_^2−^) and pH in the ocean and in seafloor pores. The model takes into account outgassing (from volcanoes and mid-oceanic ridges), continental silicate weathering, dissolution of basalts in the seafloor, and oceanic chemistry, as sources and sinks of CO_2_. For all parameters in the carbon cycle model, we use the mean values given in ref. ^5^. For the temperature dependences of silicate weathering and oceanic chemistry, we use the mean surface temperature from our climate model. Even though this model only computes global mean quantities and does not take into account the organic matter in carbon sources and sinks of carbon, it is computationally very fast and well suited for studying the major feedbacks between biological populations and the atmosphere and climate.

### Coupled biological-planetary model

The model resolves dynamics that take place on extremely different timescales—geological processes are extremely slow (10^3^–10^6^ years) compared to biological dynamics (days to years). We use a timescale separation approach and assume that the planetary environment is fixed on the biological time scale, and the biological system (population and local environment) is at ecological and evolutionary equilibrium on the geological time scale. This allows us to resolve the biological and geological dynamics separately and couple them at discrete points in time.

The biological dynamics are first resolved by the biological model for a fixed environmental forcing (equations (E11)), with microbial cells at their evolutionarily optimal size. The biological model is integrated over a sufficiently long time so that the local ecosystem reaches its equilibrium. Then we compute the biogenic fluxes between the surface ocean and the atmosphere, and between the surface ocean and the deep ocean, and feed them into the planetary model. The planetary model is then used to resolve the system’s geochemical and climate dynamics, but only for a sufficiently small change in the planetary state (1% to 1‰ variation of the state variable that changes the most) so that this change would alter the biological equilibrium only marginally. The new biological equilibrium is then computed, and the process is re-iterated. This iterative process approximates a continuous coupling between the microbial community and its planetary environment.

### Monte-Carlo simulations

We explore the parameter range by stochastically varying the parameters that determine the maximum metabolic rate, the rate of energy consumption for maintenance, the metabolic half-saturation constant, the decay and mortality rates, and the maximum division rate. We also vary the values of the parameters that shape the dependencies on size, temperature, and cellular biomass (see Supplementary Table 4). Parameter values are picked uniformly or log-uniformly (to avoid making prior assumptions on the likelihood of specific regions of the parameter space) within ranges constrained by empirical data from the literature, or large enough to cover plausible empirical variation. Three thousand simulations were run for the MG ecosystem, one thousand for the AG + AT ecosystem, and one thousand for the MG + AG + AT ecosystem, under four different values of volcanic outgassing: $\phi _{{\mathrm{Volc}}}({\mathrm{H}}_2)$ = 2 × 10^9.5^, 2 × 10^10^, 2 × 10^10.5^, and 2  × 10^11^ molecules cm^−2^ s^−1^, hence a total of 20,000 simulations. Each simulation was run to equilibrium.

The results can be divided into two subsets of roughly equivalent size, regardless of the ecosystems and volcanic activity considered: those with biological activity and those without (Supplementary Fig. 1A, B). We then verified that the number of simulations run for each of the considered scenarios was sufficient for the resulting distribution of equilibrium states to have converged. We evaluated convergence by subsampling, for each scenario, the subset of ‘biologically viable’ simulations with increasing sample size, and computing the average equilibrium biomass and CH_4_ biogenic emission of the subsamples. The results are shown in Supplementary Fig. 2 for the three scenarios and for $\phi _{{\mathrm{Volc}}}({\mathrm{H}}_2)$ = 2 × 10^10.5^ molecules cm^−2^ s^−1^.

## Supplementary information

Supplementary Information

Peer Review File

## Data Availability

The data used to produce all the results presented in this study are available upon reasonable request to B.S.

## Code availability

The codes used in this study to produce the data analyzed are available on a git repository upon reasonable request to B.S.
