# Supplementary material for: Co-evolution of primitive methane-cycling ecosystems and early Earth’s atmosphere and climate
Source: Nat Commun. 2020 Jun 1;11:2705. doi: 10.1038/s41467-020-16374-7 (PMC7264298; doi:10.1038/s41467-020-16374-7)
Supplement: Supplementary file 1 — Supplementary Information [file 41467_2020_16374_MOESM1_ESM.pdf]

Supplementary Information for

"Co-evolution of primitive methane-cycling ecosystems and early Earth's atmosphere and climate"

by Boris Sauterey et al.

Correspondence to: boris.sauterey@ens.fr

Supplementary Results and Discussion

Infuence of biological parameters on ecosystem viability

By comparing the distribution of parameter values from the subset of simulations with persistent biological activity to the distribution of all parameter values, we can delineate the region in parameter space that corresponds to ecosystem viability (Supplementary Figure 1C). We find that viability is significantly conditioned by high values of b_q, low values of basal mortality m, low values of b_E, and high values of maximum division rate r_{max}, with a predominant effect of b_q (Supplementary Figure 1D and E).

Next, we use the subset of ecologically viable simulations to examine how model parameters influence the equilibrium biomass and biogenic methane flux, $\phi_{Bio}(CH_4)$. We find that the level of biogenic methane emission is positively related to the maximum metabolic rate through parameter b_q and negatively related to the maintenance cost through b_E, while biomass production negatively correlates with both b_E and b_q(statistical analysis not shown).

Finally, we compare the distribution of outputs in the subset of ecologically viable simulations to the default parameterization outcome (Fig. 1). The distribution is relatively narrow (95% interval envelope is about one order of magnitude wide), highlighting the fact that the model is more strongly constrained by its structure than parameterization. In most scenarios, default parameter values yield results that are close to the median of the subset of ecologically viable simulations (Fig. 2). With the MG ecosystem, the results of the default parametrization are within the 95% confidence intervals, but close to the boundaries (lower limit for methane emission, upper limit for biomass; data not shown). This greater sensitivity to parameters is due to the global redox equilibrium being strongly impacted by the metabolic rate of methanogens; this is in contrast to the other ecological scenarios where the global redox equilibrium is determined chiefly by photochemical processes. The default value of q_{max} is near the lower end of the viability range for that parameter, so most of the ecologically viable simulations correspond to higher q_{max}, hence larger CH_4 emission at equilibrium and lower equilibrium biomass. This is because the redox state of the system is closer to its thermodynamic equilibrium, and therefore metabolism is less efficient. Interestingly, exploring higher values of q_{max} is equivalent to releasing kinetic constraints on biology. This explains why our predictions of CH_4 emission then get closer to ref[12].

Global redox balance of the planet

By tracking the global hydrogen budget of the atmosphere, computed as f(H_2) + 4 f(CH_4) + f(CO) following ref[6], we check the evolution of the atmospheric global redox budget in the simulations presented in Fig 4. We find that in most cases the atmospheric redox budget is very similar whether the planet is populated by a primitive biosphere or not (Supplementary Figure 11). The only two exceptions are the ecological scenarios in which AG is present in the biosphere in the absence of AT. When this is the case, some of the redox budget of the atmosphere is transferred to the ocean in the form of acetate.

The conservation of a steady atmospheric redox budget from a lifeless to a living Earth highlights that the biomass production of primitive biospheres was so low that it did not constitute a significant sink of H_2 relative to atmospheric escape (the main sink of reduced species during the Archean).

Ecological feedback of methanogenesis on climate warming and resilience

Supplementary Figure 3 shows the atmospheric and climatic impact of the biosphere as a function of H_2 volcanic outgassing, $\phi_{Volc}(H_2)$, and the abiotic temperature, T_{Geo}. Here T_{Geo} varies independently of pCO_2 due to external factors such as solar activity. Two values of pCO_2 are tested: 2500 ppm (Supplementary Figure 3A) to allow comparison with ref[12], and 10^5 ppm (Supplementary Figure 3B) for comparison with Fig. 1 and 3 (the climate model predicts 10^5 ppm CO_2 to set T_{Geo} at 12 °C). Qualitatively, the results are similar to those reported in Fig. 3 where temperature is set by pCO_2. Quantitatively, with the MG ecosystem the effect of temperature variation on biological activity is even stronger when T_{Geo} and pCO_2 are independent. This is because the negative effect of higher temperature on thermodynamics is partially offset if pCO_2 is concomitantly higher. As a consequence, hydrogenotrophic methanogenesis is expected to have an even greater effect on climate when temperature varies independently of pCO_2. In contrast, climate warming by AG+AT metabolisms is weak, irrespective of H_2 outgassing and abiotic temperature when the latter varies independently of pCO_2. Warming will occur, however, in an AG+AT ecosystem in which MG evolves, the effect being as strong as in the MG-only ecosystem (Supplementary Figure 3A).

Climate resilience to variation of pCO_2 is shown in Supplementary Figure 4. With the MG ecosystem, the ecological feedback to the atmosphere has a buffering effect on temperature variation above *ca.* 5 °C (Supplementary Figure 4A) and an amplifying effect below 5 °C (Supplementary Figure 4B). With the AG+AT ecosystems, the amplification effect prevails irrespective of the temperature range (Supplementary Figure 4C and D). Once MG, AG and AT have all evolved, we can however conclude from Fig. 3 that the ecosystem has almost no effect on the resilience of the climate.

Atmospheric and climatic impact of methanotrophy

CH_4 emissions by MG and AG+AT metabolisms create conditions favorable for the evolution of methanotrophy (MT). The evolutionary rate has a critical influence on the MT environmental feedback. Supplementary Figure 6 shows the environmental impact of fast-evolving MT that arises on a 10^3 years timescale after the establishment of MG and/or AG+AT metabolisms. In this case, MT evolution takes place under atmospheric and climatic conditions set by the atmosphere-ecosystem equilibrium of MG and/or AG+AT (Figs. 2 and 3, Supplementary Figure 3). Irrespective of H_2 volcanic outgassing rate and abiotic temperature (Supplementary Figure 6A), the environmental effect of MT is to consume most of the atmospheric CH_4 produced by methanogens (Supplementary Figure 6C), driving the surface temperature close to its abiotic value, T_{Geo} (Supplementary Figure 6B). The timescale over which this happens is very short, of the order of 10^3 years (Supplementary Figure 6B

and C). The outcome is a new atmosphere-ecosystem equilibrium at which all metabolisms coexist, under a methane-poor atmosphere resulting in a cool climate.

The previous scenario will hold provided the evolutionary timescale is much shorter than the timescale of the carbon cycle. If the timescale of MT evolution is of the order of the C cycle characteristic time (10^7 yrs), or longer, then the environmental impact of MT evolution will depend on the long-term effect that the carbon cycle has on the environment inhabited by MG and/or AG+AT ecosystems (Supplementary Figure 12). As explained in the main text, the carbon cycle response to the evolution of methanogenesis leaves the biogenic outflux of CH_4 relatively unaltered. However, the equilibrium temperature, T_{BioGeo}, is much lower than at the short-term equilibrium shown in Fig. 3, in the absence of carbon cycle feedback. Under such conditions, the evolution of methanotrophy drives both pCH_4 and pCO_2 down (Fig. 5A), causing dramatic climate cooling and putting the planet at high risk of global glaciation (Fig. 5B and C). Supplementary Figure 8C shows that relatively low abiotic temperature combined with a high rate of H_2 volcanic outgassing favors the global glaciation outcome.

Supplementary Figures

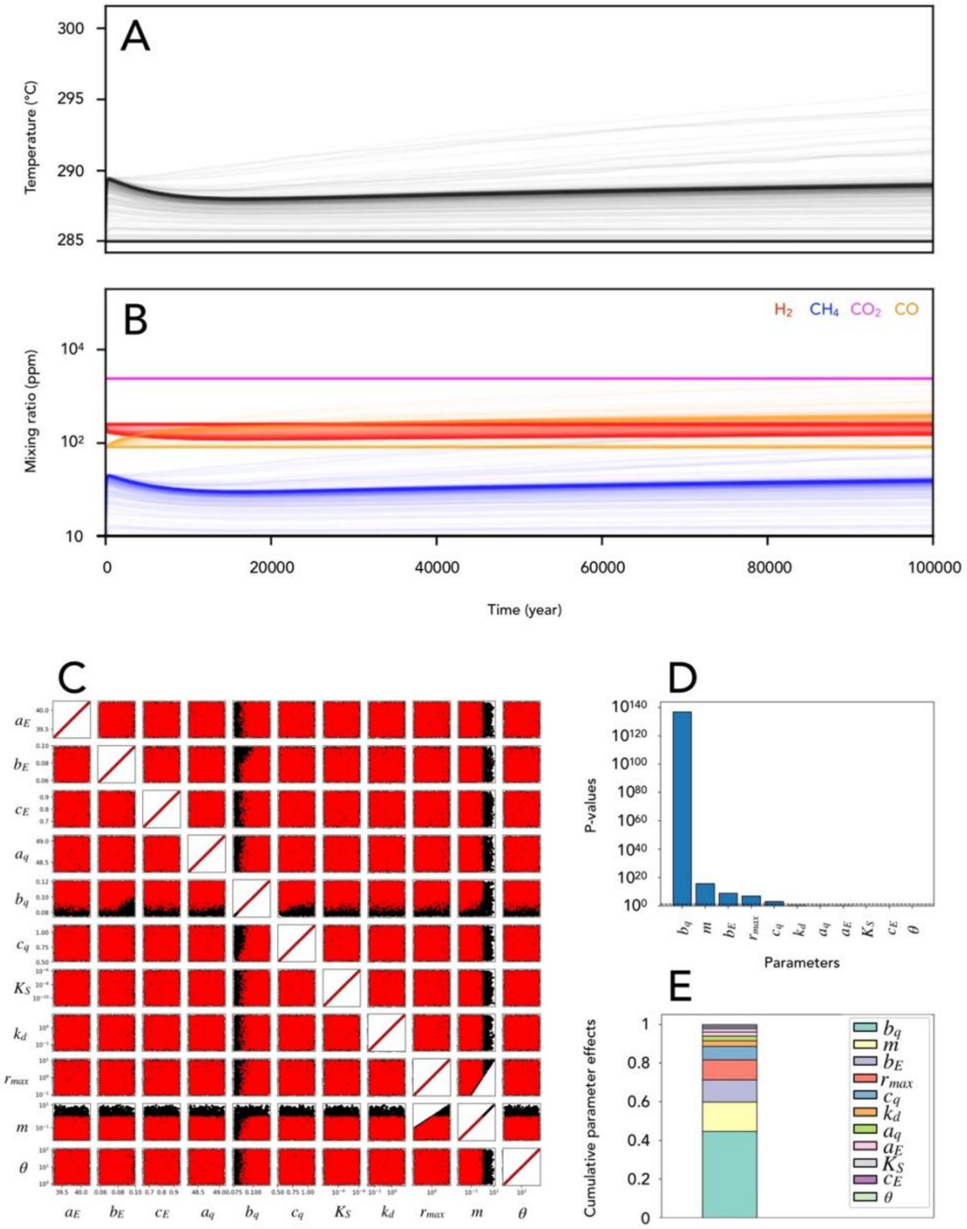

Supplementary Figure 1.
Outputs of 5,000 Monte Carlo simulations for the MG ecosystem. (**A**) Surface temperature. (**B**) Atmospheric composition. (**C**) Ecosystem viability across the parameter space. Red dots indicate simulations in which the ecosystem is viable; other simulations are indicated in black. A discrepancy between the distributions of black and red dots for a given parameter indicates that viability is favored by a specific range in that parameter's value. The significance and strength of each parameter's influence on MG ecosystem viability is given in panels **D** and **E**, respectively. Volcanic H_2 outgassing $\phi_{Volc}(H_2)$ is set to 2 $10^{9.5}$, other parameters are set to their default values (Supplementary Tables 2 and 3).

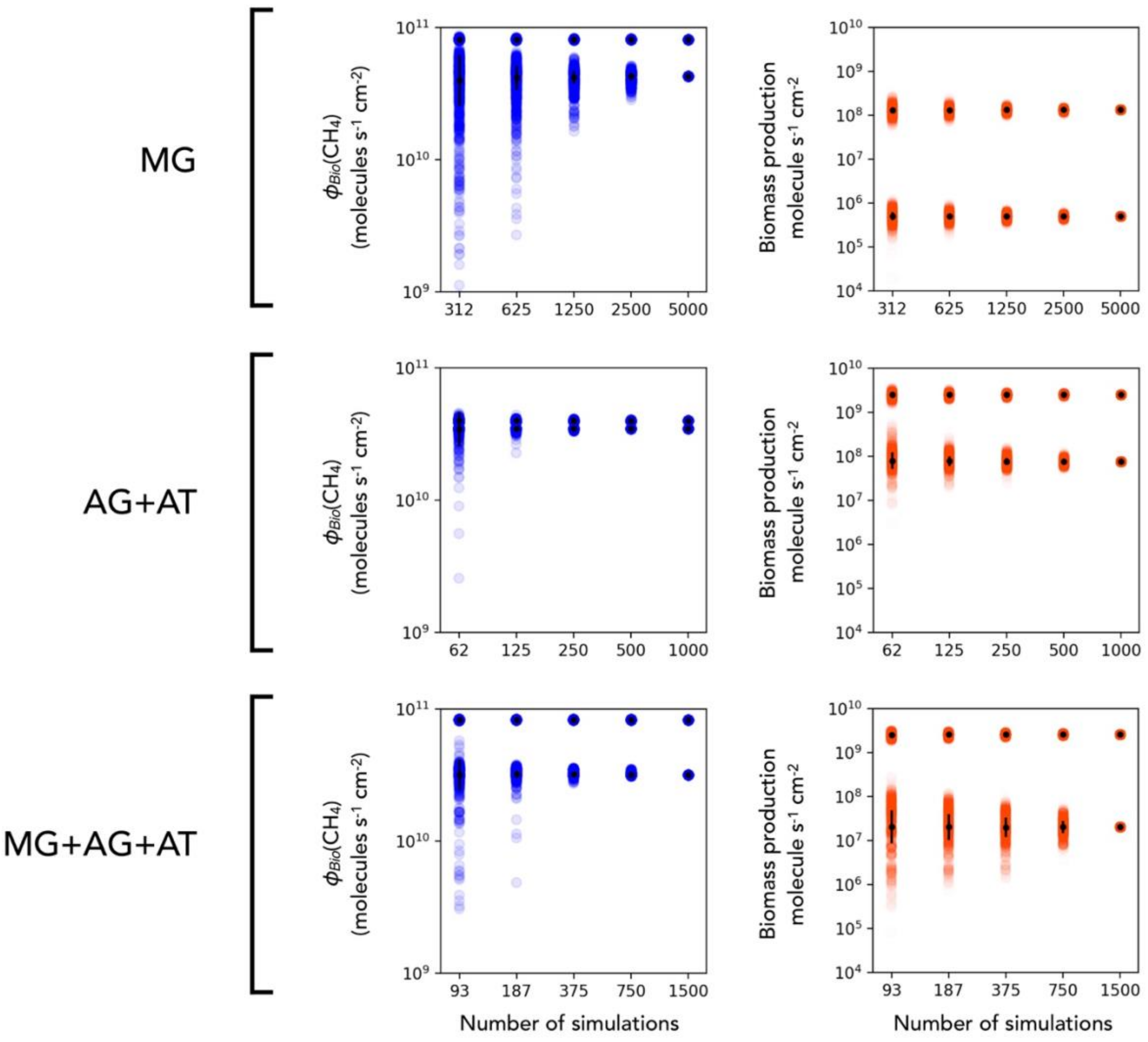

Supplementary Figure 2.

Influence of the number of simulations on the higher and lower boundaries of biomass production and biogenic emission of CH4, $\phi_{Bio}(CH_4)$. The numbers of simulations represent 1/16, ⅛, ¼, ½ and the full set of the whole simulations ensemble. For each simulation set size, a thousand subsamples of that size are bootstrapped, for which the average value (dot) and standard deviation (error bar) are calculated. Noticingly, for all three types of ecosystems, the lower boundary is largely underestimated when the number of simulations is too low, and converge toward its actual value as the number of simulations increases. H_2 volcanic outgassing is fixed atof 2 $10^{10.5}$ molecules cm^{-2} s^{-1}, other parameters are set to their default values (Supplementary Tables 2 and 3).

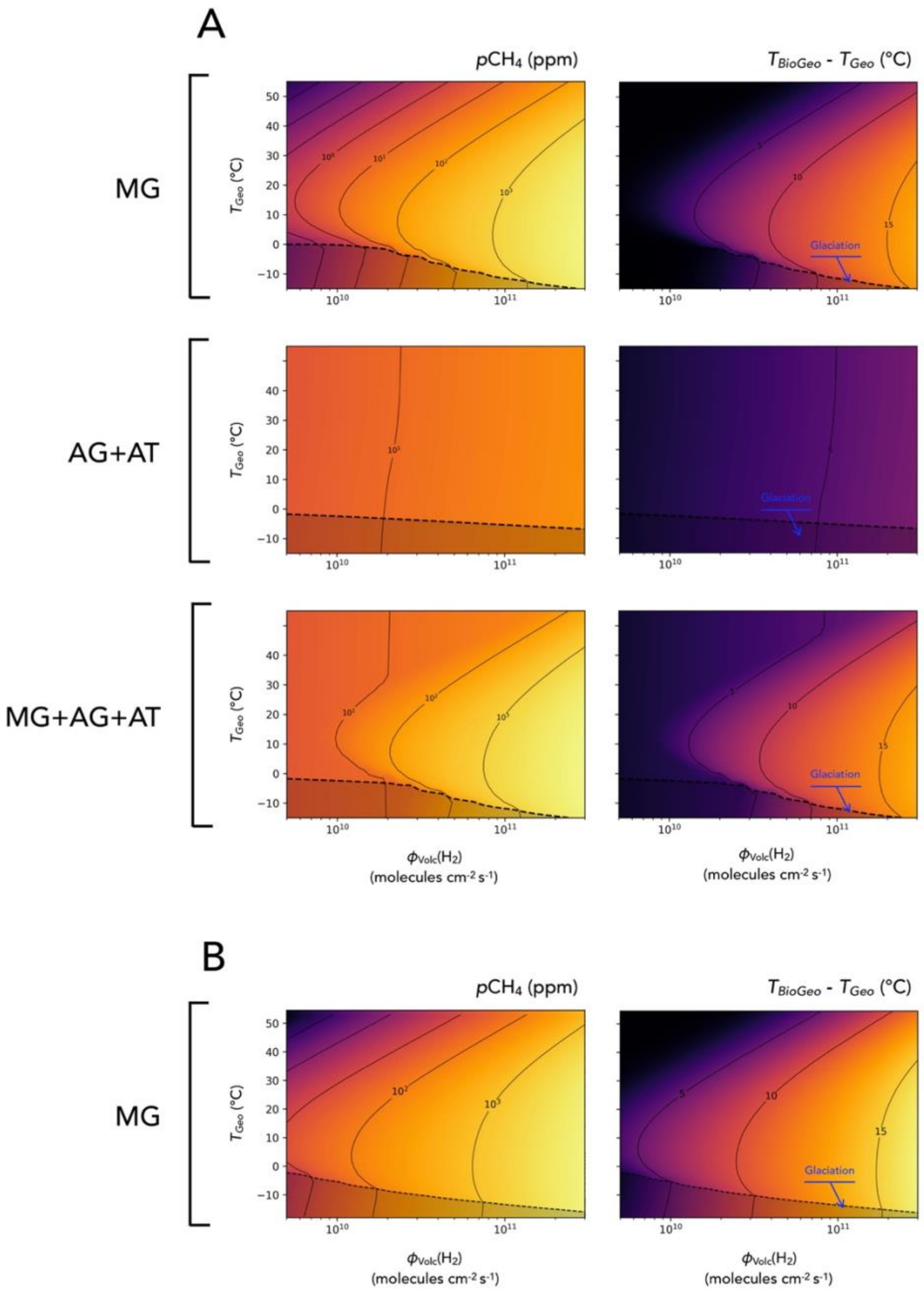

Supplementary Figure 3.

Biogeochemical response of the early Archean Earth to changes in H_2 volcanic outgassing, abiotic temperature (T_{Geo}) and ecosystem composition. Here T_{Geo} is varied independently of pCO_2 in the climate model. (A) pCO_2 = 2500 ppm. (B) pCO_2 = 10^5 ppm. *Left*, Atmospheric pCH_4 at ecosystem-climate equilibrium. Shaded areas indicate conditions for organic haze formation. *Right*, Temperature differential between T_{Geo} and the global surface temperature reached at ecosystem-climate equilibrium, T_{BioGeo} . Shaded areas indicate conditions leading to glaciation. Other parameters are fixed to their default values (Supplementary Tables S2 and S3).

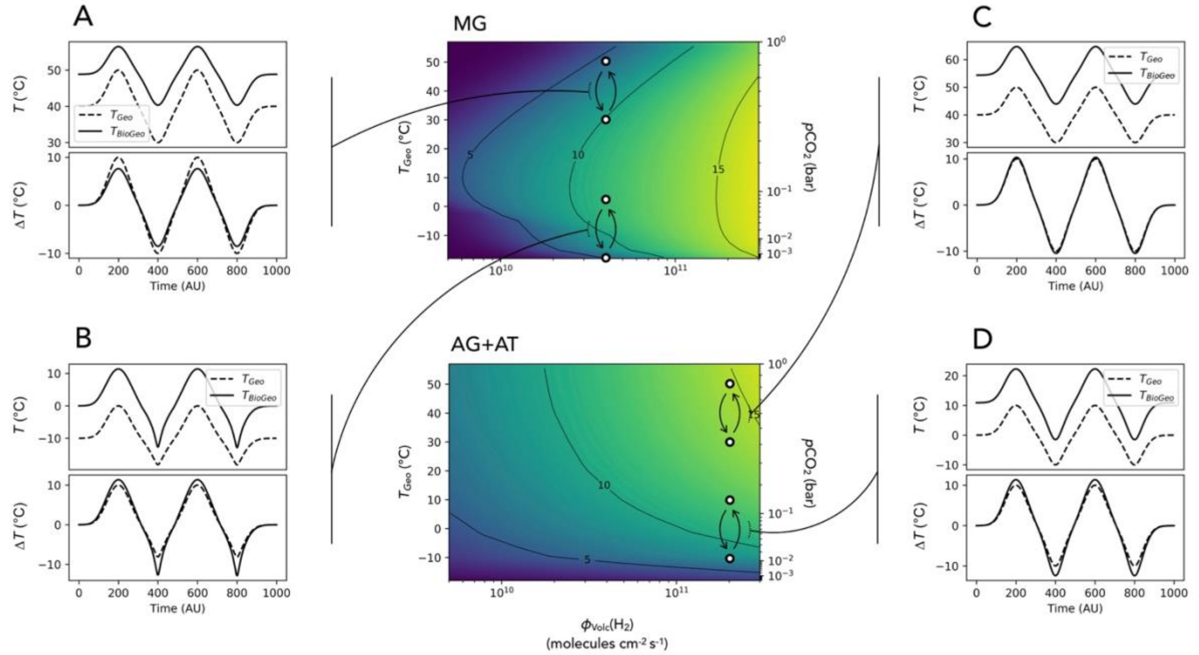

Supplementary Figure S4.

Climate resilience in response to pCO_2 variation. The central color panels are from Fig. 3. Side panels **A**-**D** show the climatic response of the planet, either inhabited (T_{BioGeo}, plain curves) or lifeless (T_{Geo}, dashed curves), to periodic variation in pCO_2, depending on ecosystem composition (MG or AG+AT). The corresponding abiotic temperature variation amplitude is ΔT_{Geo} = 20 °C (indicated by the white dots and arrows in the central color panels). On a warm planet inhabited by MG (T_{Geo} ranging from 30 to 50 °C), the climate response is buffered by about 20 % (A). However, on a cool planet (T_{Geo} ranging from -20 to 0 °C, panel) the MG ecosystem amplifies the climate response by up to 33% (B). On a planet inhabited by AG+AT, the climate response to pCO2 variation is always amplified, but much less on a warm planet (C) (5% for T_{Geo} ranging from 30 to 50 °C) than on a cool planet (33% for T_{Geo} ranging from -10 to 10 °C, bottom-right panel) (D).

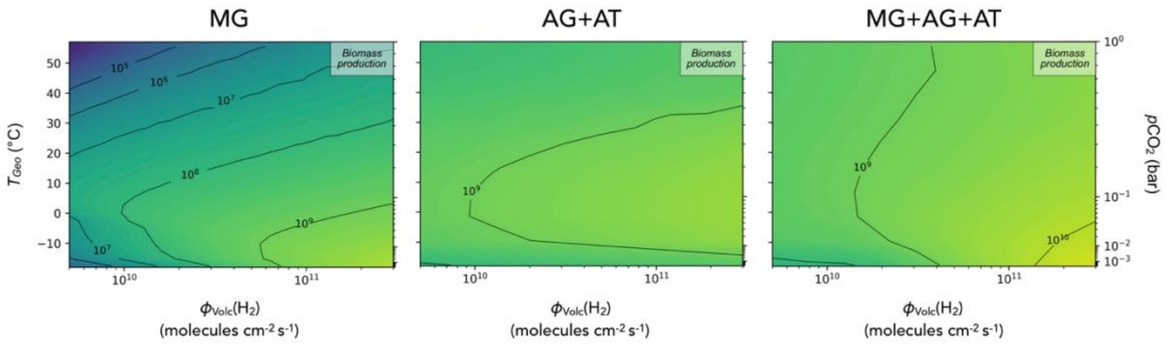

Supplementary Figure 5.
Equilibrium biomass production (in molecules C cm^{-2} s^{-1}) for each ecosystem composition. The abiotic surface temperature, T_{Geo}, is determined by $p CO_2$. Other parameters are set to their default values (Supplementary Tables 2 and 3).

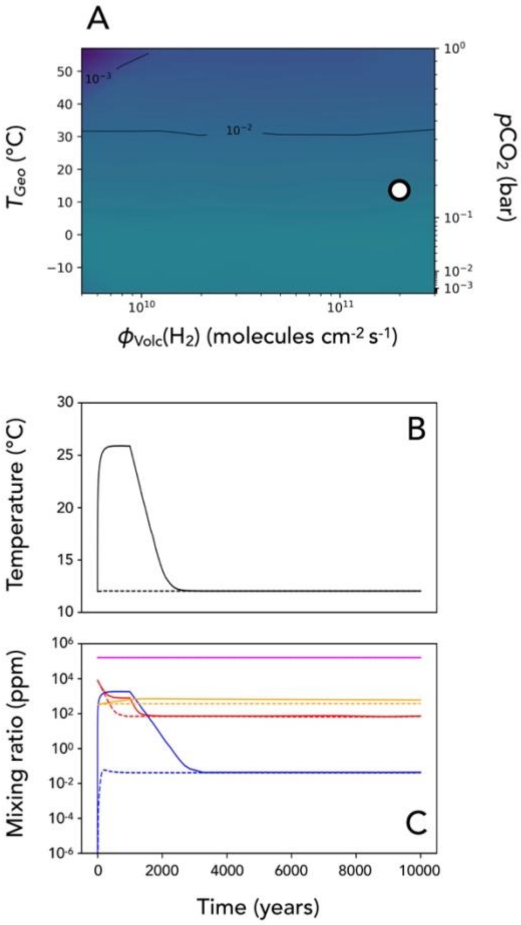

Supplementary Figure 6.

Biogeochemical response of the early Archean Earth as MT evolves into the MG ecosystem. (**A**) Effect of H_2 volcanic outgassing and abiotic temperature, T_{Geo}, on atmospheric pCH_4 at ecosystem-climate equilibrium. Here T_{Geo} is determined by pCO_2. (**B**, **C**) Effect of MT evolving with MG (dotted lines) or 1,000 years after MG (plain lines) on temperature (B) and mixing ratios (C) of CH_4 (blue), H_2 (red), CO (yellow) and CO_2 (magenta), for T_{Geo} and $\phi_{Volc}(H_2)$ indicated by the white dot in (A). All other parameters are fixed at their default values (Supplementary Tables 2 and 3).

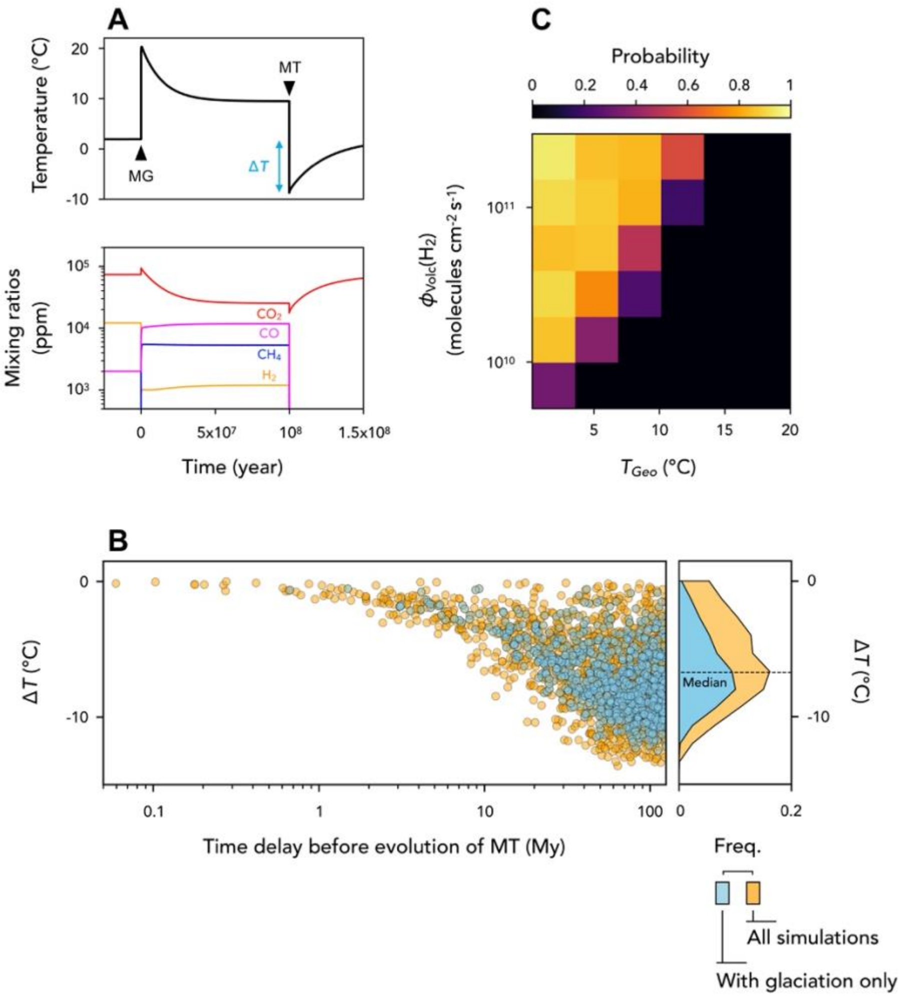

Supplementary Figure 7.
Climatic and atmospheric destabilization by evolutionary metabolic innovation (methanotrophy). (**A**) Example with T_{Geo} = 2 °C, $\varphi_{volc}(H_2)$ = 3 10^{11} molecules s^{-1} cm^{-2}, and sulfur-based methanotrophs (MT) evolving 100 million years after MG (instead of MG-AG-AT as in the main text). *Top*, Change in surface temperature. *Bottom*, Change in atmospheric composition. Panels (**B**) Distribution of outcomes across a range of abiotic temperature T_{Geo}, H_2 volcanic flux, and evolution time of MT (2,000 randomly chosen combinations). *Left*, Amplitude of global cooling, ΔT, with respect to the evolution time of MT. *Right*, Frequency distribution of all temperature changes ΔT (blue) and of temperature changes conditional on glaciation outcome (yellow). (**C**) Estimated probability of glaciation as a consequence of MT evolution, given the abiotic temperature T_{Geo} and H_2 volcanic flux. Other parameters are set to their default values (Supplementary Tables 2 and 3).

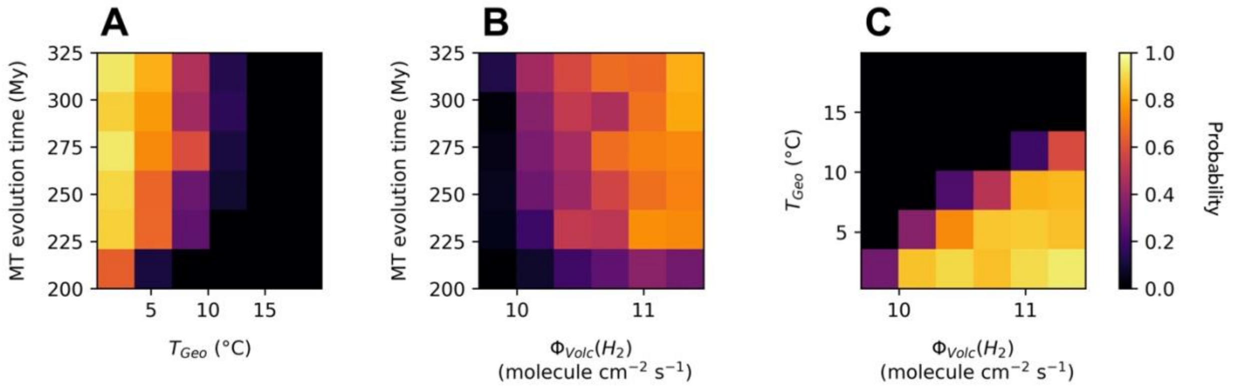

Supplementary Figure 8.
Probability of climate destabilization (global cooling leading to glaciation) by the evolution of methanotrophy (MT). Influence of three key parameters: (**A**) Abiotic temperature, T_{Geo}, and MT evolution time; (**B**) H2 volcanic outgassing, $\phi_{Volc}(H_2)$**,** and MT evolution time; (**C**) H2 volcanic outgassing, $\phi_{Volc}(H_2)$, and abiotic temperature, T_{Geo}.

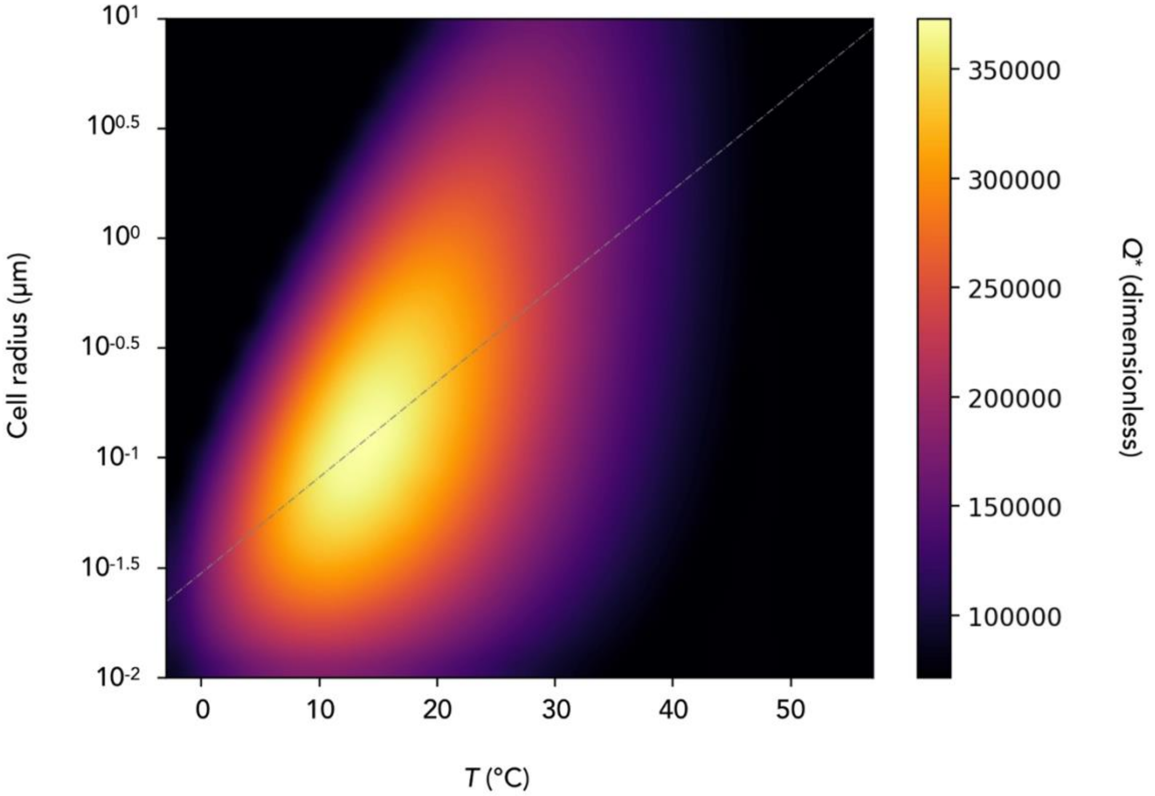

Supplementary Figure 9.
Equilibrium thermodynamic reaction quotient, *Q, (inversely correlated to resource use) as a function of cell radius and temperature.** The dashed line indicates the evolutionarily optimal cell size, i.e., cell size corresponding to the highest Q*, as a function of temperature. Parameters are set to their default values (Supplementary Tables 2 and 3).

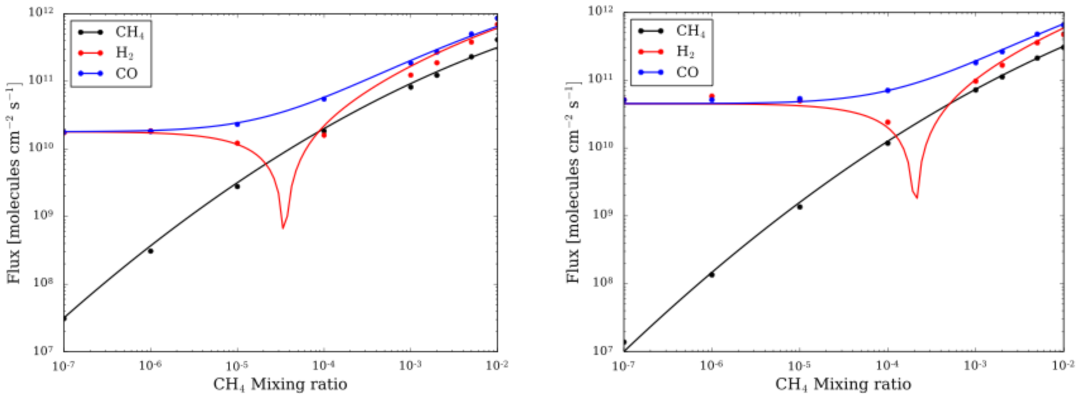

Supplementary Figure 10.

Absolute net flux of CH_4, H_2 and O_2 due to CO_2 and CH_4 photolysis as a function of the CH_4 mixing ratio with 0.1 bar of CO_2, 100 ppm (*left*) and 1000 ppm of H_2 (*right*). Dots correspond to the results of the 1D photochemical model and lines to our parameterization. The photochemistry leads to a production of CO and destruction of CH_4 in all cases. For low mixing ratios of CH_4, the H_2 flux is negative by the photolysis of CO_2. For high mixing ratios of CH_4, the H_2 flux is positive due to the photolysis of CH_4.

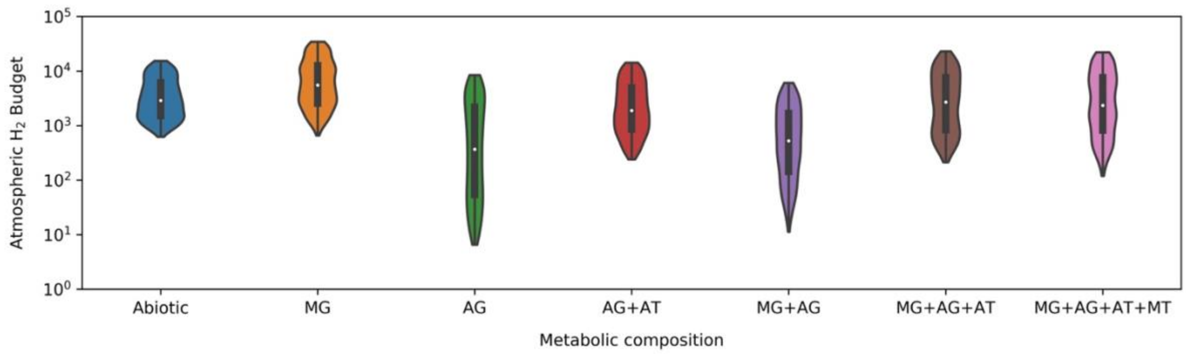

Supplementary Figure 11.

Evolution of the atmospheric global redox budget evaluated as the atmospheric H_2 budget (in ppms) for each biosphere composition. The global hydrogen budget of the atmosphere is computed as f(H_2) + 4 f(CH_4) + f(CO) following ref[6]. Results derived from the simulations presented in Fig. 4 (1,000 simulations in each scenario), obtained by drawing uniformly the model abiotic parameter values in log-uniform priors based on the litterature (see Table 1). The white dots represent the median of the distributions, the thick gray lines the interquartile range, and the thin gray lines the rest of the distribution.

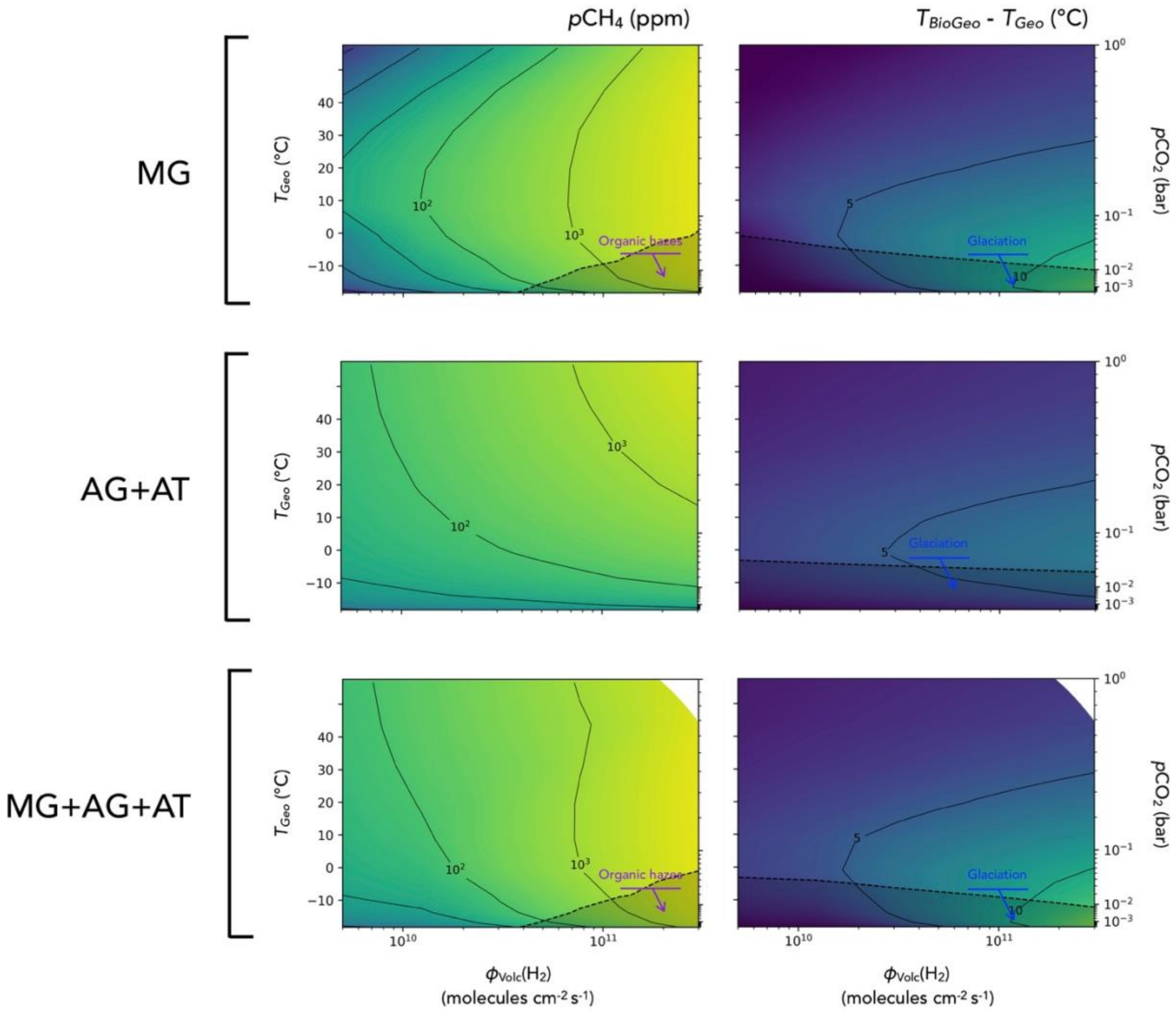

Supplementary Figure 12.

Long-term effect of the carbon cycle on the biogeochemical response of the early Archean Earth to changes in H_2 volcanic outgassing, abiotic temperature (T_{Geo}) and ecosystem composition. Here T_{Geo} is determined by pCO_2 in the climate model. *Left*, Atmospheric pCH_4 at ecosystem-climate equilibrium. Shaded areas indicate conditions for organic haze formation (pCH_4:pCO_2 > 0.2). *Right*, Temperature differential between T_{Geo} and the global surface temperature reached at ecosystem-climate equilibrium, T_{BioGeo}. Shaded areas indicate conditions leading to glaciation ($T_{BioGeo} < 0°C$). Other parameters are fixed to their default values (Supplementary Tables 2 and 3).

Supplementary Table 1.
Metabolic reactions and their thermodynamic constants.

Name	Notation	Catabolic reaction	ΔG0	ΔH0
H_2-based methanogens	MG	$4 \cdot H_2 + CO_2 \rightarrow CH_4 + 2 \cdot H_2O$	-32.6 kJ.mole--1	-63.2 kJ.mole--1
Sulfur-based methanotrophs	MT	$CH_4 + H_2SO_4 \rightarrow H_2S + CO_2 + 2 \cdot H_2O$	-107 kJ.mole--1	-1.8 kJ.mole--1
Acetogens	AG	$4 \cdot CO + 2 \cdot H_2O \rightarrow CH_3COOH + 2 \cdot CO_2$	-77.9 kJ.mole--1	-129.9 kJ.mole--1
Acetotrophs	AT	$CH_3COOH \rightarrow CH_4 + CO_2$	-55 kJ.mole--1	16.2 kJ.mole--1
Shared anabolic reaction:		$10 \cdot CO_2 + N_2 + 24 \cdot H_2 \rightarrow C_{10}H_{18}O_5N_2 + 1.5 \cdot H_2O$	28.25 kJ.mole--1	128 kJ.mole--1

Supplementary Table 2.
Default parameter values in the biological model.

Parameter (and reference)	Notation	Value or expression	Unit
Cell radius	r_C	$10^{a_r+b_r \cdot T}$	μm
Cell volume	V_C	$\frac{4}{3} \cdot \pi \cdot {r_c}^3$	μm^3
Structural carbon content	B_{Struct}[17]	$18 \cdot 10^{-15} \cdot {V_C}^{0.94}$	$mol\ C\ cell^{-1}$
Maximum metabolic rate	q_{max}	$e^{a_q+b_q \cdot T} \cdot {V_C}^{c_q}$	d^{-1}
	a_q[18]	-55.76	
	b_q[19]	0.1	
	c_q[21,22]	0.82	
Half-saturation constant	K_S	10^{-9}	$mol. L^{-1}$
Maintenance rate	E_m	$e^{a_E+b_E \cdot T} \cdot {V_C}^{c_E} \cdot 10^{-3}$	$kJ. d^{-1}$
	a_E[18]	-43.54	
	b_E[20]	0.08	
	c_E23	0.67	
Decay rate	k_d	0.5	d^{-1}
Basal mortality rate	m	0.1	d^{-1}
Maximum division rate	r_{max}	1	d^{-1}
Division rate dependence on internal reserve	θ	10	dimensionless

Supplementary Table 3.
Default values for parameters in metabolism specific size dependency on temperature.

Metabolism	a_r	b_r
MG	-13.23	0.0431
MT	-13.289	0.0432
AG	-13.21	0.044
AT	-12.55	0.042

Supplementary Table 4.
Ranges of biological parameter values used in Monte-Carlo simulations.

Parameter	Symbole	Range explored	Prior distribution
Maximum metabolic rate	a_q	-55.20 – -56	uniform
	b_q	0.076 – 0.12	uniform
	c_q	0.53 – 1.10	uniform
Half-saturation constant	K_S	10^{-11} – 10^{-6}	log-uniform
Maintenance rate	a_E	-43.03 – -43.93	uniform
	b_E	0.059 – 0.098	uniform
	c_E	0.66 – 0.94	uniform
Decay rate	k_d	0.05 – 5	log-uniform
Basal mortality rate	m	0.01 – r_{max}	log-uniform
Maximum division rate	r_{max}	0.1 – 10	log-uniform
Division rate dependence on internal reserve	θ	1 – 100	log-uniform

Supplementary References

1. Catling, David C., and James F. Kasting. *Atmospheric evolution on inhabited and lifeless worlds*. Cambridge University Press, 2017.

2. Kharecha, P., J. Kasting, and J. Siefert. "A coupled atmosphere–ecosystem model of the early Archean Earth." *Geobiology* 3.2 (2005): 53-76.

3. Kleerebezem, Robbert, and Mark CM Van Loosdrecht. "A generalized method for thermodynamic state analysis of environmental systems." *Critical Reviews in Environmental Science and Technology* 40.1 (2010): 1-54.

4. González-Cabaleiro, Rebeca, Juan M. Lema, and Jorge Rodríguez. "Metabolic energy-based modelling explains product yielding in anaerobic mixed culture fermentations." *PLoS One* 10.5 (2015): e0126739.

5. Tijhuis, L., Mark CM Van Loosdrecht, and J. J. Heijnen. "A thermodynamically based correlation for maintenance Gibbs energy requirements in aerobic and anaerobic chemotrophic growth." *Biotechnology and bioengineering* 42.4 (1993): 509-519.

6. Litchman, Elena, et al. "The role of functional traits and trade-offs in structuring phytoplankton communities: scaling from cellular to ecosystem level." *Ecology letters* 10.12 (2007): 1170-1181.

7. Ward, Ben A., et al. "The size dependence of phytoplankton growth rates: a trade-off between nutrient uptake and metabolism." *The American Naturalist* 189.2 (2017): 170-177.

8. Gillooly, James F., et al. "Effects of size and temperature on metabolic rate." *science* 293.5538 (2001): 2248-2251.

9. Aksnes, D. L., and J. K. Egge. "A theoretical model for nutrient uptake in phytoplankton." *Marine ecology progress series. Oldendorf* 70.1 (1991): 65-72.
