# Supplementary material for: Co-evolution of primitive methane-cycling ecosystems and early Earth’s atmosphere and climate
Source: Nat Commun. 2020 Jun 1;11:2705. doi: 10.1038/s41467-020-16374-7 (PMC7264298; doi:10.1038/s41467-020-16374-7)
Supplement: Supplementary file 2 — Peer Review File [file 41467_2020_16374_MOESM2_ESM.docx]

Peer Review Information

**Journal:** Nature Communications

**Manuscript title:** Co-evolution of primitive methane-cycling ecosystems and early Earth’s atmosphere and climate

**Corresponding author name(s):** Dr Boris Sauterey

**Editorial notes:**

**Transferred manuscripts**

This manuscript has been previously reviewed at another journal that is not operating a transparent peer review scheme. This document only contains reviewer comments and rebuttal letters for versions considered at *Nature Communications*.

**Transferred manuscripts (no peer review at *Nature Communications*)**

This manuscript has been previously reviewed at another journal that is not operating a transparent peer review scheme. The manuscript was considered suitable for publication without further review at *Nature Communications*.

**Redactions – transferred manuscripts (mention of previous referee reports from elsewhere)**

This manuscript has been previously reviewed at another journal that is not operating a transparent peer review scheme. This document only contains reviewer comments and rebuttal letters for versions considered at *Nature Communications*. Mentions of prior referee reports have been redacted.

**Redactions – transferred manuscripts (mention of the other journal)**

This manuscript has been previously reviewed at another journal that is not operating a transparent peer review scheme. This document only contains reviewer comments and rebuttal letters for versions considered at *Nature Communications*. Mentions of the other journal have been redacted.

**Redactions – unpublished data**

Parts of this Peer Review File have been redacted as indicated to maintain the confidentiality of unpublished data.

**Redactions – confidential patient information**

Parts of this Peer Review File have been redacted as indicated to maintain patient confidentiality.

**Redactions – published data**

Parts of this Peer Review File have been redacted as indicated to remove third-party material where no permission to publish could be obtained.

**Amendments – published data**

[Figure X/The figure on page X] in this Peer Review File has been amended to remove third-party material where no permission to publish could be obtained.

**Published data (credit line for image)**

[Figure X/The figure on page X] in this Peer Review File is reproduced with permission from [Copyright Holder].

**Redactions – reviewer opt-out**

Parts of this Peer Review File have been redacted as indicated as we could not obtain permission to publish the reports of reviewer ###.

**Reviewer comments in marked-up manuscript**

In their review of the [first/second/third/...] version of this manuscript, reviewer ### added their comments to the manuscript file. These comments, excluding minor textual revisions, have been copied into this Peer Review File.

**Reviewer comments & decisions:**

**Reviewer comments, first version:**

Reviewers' comments:

Reviewer #1 (Remarks to the Author):

The paper by Sauterey et al. models the changes in the climate of the early Earth in response to the evolution of methanogenesis, acetogenesis and methanotrophy. The question is of general interest with important implications on the evolution of life on the early Earth as well as on other planets.

While I am not familiar enough with modelling to evaluate that aspect of the paper, the microbiology appears to be sound.
Some questions that should be addressed and other suggestions are:

Figure 2. The meaning of the right hand panel is not clear. For instance, how can biomass production have units of ‘molecules cm-2 s-1’? Molecules of what?

Line 29. Rewrite as: “…function preceded the evolution of phototrophs.”

Line 41. Substitute “to model” with “when modelling”

Line 50-51. The definition of methanogens is incorrect. Most organisms use “redox potential” as an energy source. The defining character is the electron acceptor, which is CO2 for methanogens. Please rewrite as “(unicellular organisms that couple methane biosynthesis with growth)”

Lines 103-105. This sentence seems to equate biomass production with the amount of standing biomass, which is recorded in the geological record. The amount of standing biomass also depends on the rate of degradation, presumably by heterotrophic prokaryotes. If degradation is slow, the standing biomass can be quite large even with slow production rates.

Lines 167-174. Figure 4C seems to state that the probability of glaciation increases with higher fluxes of H2. Since H2 flux only increases MG, why doesn’t it lead to even small methane increases and some warming?

Line 198. “other Earths”. Please rewrite. There is only one Earth.


Reviewer #2 (Remarks to the Author):

The authors provide results of a coupled biology-planetary model to estimate the influence of a pre-photosynthetic prokaryotic community on planetary habitability. They relate these findings to certain metabolic innovations on earth as well as the potential of chemolithotrophic communities on early earth. This work is novel in that it combines several models, multiple simulations, with statistical evaluations in an effort to describe the earliest carbon cycle on earth. They investigate three different ecosystems, all methane based, and describe the impact each of those would have on planet habitability. This is relevant to the search for life on Mars, of extrasolar planets, as well as our general understanding of how life originated on our planet. The authors also make a strong case that their results give credence or the value of the innovation of photosynthetic metabolism on earth’s habitability. Conceptually, the project is a difficult one. While it is not novel in concept (Kasting et al, referenced in manuscript), it is indeed a novel and vastly improved effort from previous attempts. And it certainly contributes in a substantial way to our understanding that a very primitive community of methane producing and consuming individuals was likely not sufficient to provide climate stability early on. This is satisfying in light of our current assessment of very ancient stromatolitic communities (Allwood, et al). (That is, these early stromatolite fossils were formed through a complex community of organisms whose constituency included photosynthetic members. This corroborates the author’s suggestion that the timing of metabolic innovation was critical in earth’s history early on.) There is application of their model protocols for additional refinements as well as other applications. I would consider the authors’ efforts as valuable for continued research not only in climate and geology but in environmental microbiology. I have heard it said in geology circles that microbial interactions are minimal when regarding the time scale that on which geological change operates. This research offers a different view that as a microbiologist I find satisfying. Not only does it model and fit microbial community dynamics to surface geochemistry and climate despite time scale differences, it posits that it might not even be necessary to have a large microbial biomass present to significantly impact planet habitability. These kinds of insights are the kind that influence thinking across multiple disciplines. Also, there is sufficient detail provided that other researchers could utilize the models and protocols reported here in. I find that modelers of climate and geology are researchers who tend to share code. This report exhibits that to be sure in the method details provided as well as the researchers they have acknowledged.


Reviewer #3 (Remarks to the Author):

Review of “Co-evolution of primitive methane-cycling ecosystems and early Earth habitability”

This paper presents a new approach to understanding biogeochemical cycles on the early Earth. A detailed model of chemolithoautotrophic ecosystems is developed that captures microbial population dynamics by considering the thermodynamic and kinetic limitations to cell growth. This ecosystem model is coupled to parameterized climate, carbon cycle, and photochemical models to investigate the ways in which biological feedbacks and evolutionary innovations could modulate planetary habitability. The key findings are that (i) biological methane cycling by non-photosynthetic ecosystems can strongly affect atmospheric composition despite total biomass being low, (ii) biological methane cycling has an anti-Gaian tendency to amplify geologically driven temperature perturbations, and (iii) the evolution of methanotrophy may have resulted in dramatic global cooling.

The novel modeling approach in this paper is impressive. By explicitly incorporating the thermodynamic and kinetic limitations on metabolism and self-consistently modeling cell growth, catabolism, anabolism, and cell death, the ecosystem model seemingly has the potential to explore biogeochemical interactions much more rigorously than has been attempted previously. The authors ought to be commended for this innovative approach.

However, I feel as though the paper is incomplete in its current form. Although I can see the potential of this kind of detailed modeling, the paper does not clearly demonstrate how and why their model makes different predictions to previous models of methane-cycling ecosystems. The significant complexity added via ecosystem modeling requires further justification. Moreover, the novelty of the main results needs to be better highlighted and quantified. The idea that methane cycling in non-photosynthetic ecosystems strongly affects atmospheric composition, and therefore climate, is not new (e.g. Kharecha et al. 2005). Similarly, the idea that evolutionary innovations such as the emergence of methanotrophy could trigger glaciations is fairly unsurprising. This paper requires major revisions to better highlight novel conclusions and justify the modeling approach. Methodologies and assumptions also need to be clarified at several points in the manuscript.

Specific comments:

- The abstract states that “How primitive, non-photosynthetic ecosystems influenced the early habitability of Earth is poorly understood. A full understanding of this issue requires models that couple the ecological function and biological evolution of chemolithotrophic ecosystems and their interactions with the planet surface geochemistry and climate.” The paper ought to do more to convince the reader that these ecological models provide new insights compared to previous models where ecosystems are represented by net reactions added to photochemical models (e.g. Kharecha et al. 2005, Geobiology; Schwieterman et al. 2019, ApJ). For example, how do biomass production outputs in Fig. 2 compare to those for the same metabolisms in Kharecha et al. 2005? An explicit graphical comparison would be helpful. It looks like the estimates in this study are considerably lower. Why is this the case?

- The main text assumes that methanotrophy evolved shortly after methanogenesis, but it could be argued that methanotorphy probably didn’t emerge until after oxygenic photosynthesis due to lack of viable oxidants in the Archean ocean. There is some discussion of this issue in the supplementary materials, but I think this needs to be explored in the main text since it underlies Fig. 4.

- On a related note, I am unsure as to whether this paper’s estimates of the Archean sulfate reservoir are reasonable. For example, Crowe et al. (2014; Science) estimate Archean sulfate concentrations to be <2.5 micromolar (c.f. 4 mM assumed in this study). Habicht et al. (2002; Science) estimate concentrations of <0.2 mM. Admittedly, these estimates assume some form of biological sulfate reduction, but microbial sulfate reduction could conceivably use reductants other than methane.

- The 4 mM oceanic concentration used in this study seems to come from a 7.2e12 L/yr flux of water through hydrothermal systems, and an assumption that all sulfate is removed in these high-temperature systems. But this is not necessarily the only significant sulfate sink in the reducing Archean environment. Low-temperature aqueous reactions and/or evaporative sinks could contribute, leading to a smaller sulfate reservoir.

- The paper implicitly assumes that life is not nutrient limited. This assumption needs to be made explicit and justified, with appropriate caveats. Some estimates of Archean phosphorus abundances in the ocean suggest concentrations 3-5 orders of magnitude less than in the modern ocean (e.g. Kipp and Stueken 2017; Science Advances, and references therein). This overlaps with the Archean biomass estimates in this paper (4-7 orders of magnitude lower than modern), and so the possibility of phosphorus limited non-photosynthetic biospheres cannot be excluded. Nitrogen limitation presumably also cannot be ruled out unless it has been established that biological nitrogen fixation predates methanogenesis and acetogenesis.

- Some discussion of the likely order of emergence of methanogenesis vs acetogenesis/acetotrophy would be helpful. Would a world with methanogens but no acetogens persist for very long? Presumably the CO-accumulation that results from a methanogen-only ecosystem would be a strong evolutionary driver for CO-consuming acetogens? What do LUCA reconstructions have to say on the subject?

- On a related note, could the model outputs from this study be used to make some quantitative, testable predictions about the Archean atmosphere’s composition through time? Recent studies have helped constrain the hydrogen and methane abundances of the early Earth (e.g. Kadoya and Catling, 2019, GCA; Zahnle et al. 2019, GCA). More precise proxies will surely follow. In my mind, quantitative predictions of atmospheric composition would be more useful than the climate perturbation/glaciation calculations in Fig. 4 since the latter will be modulated by uncertain paleogeography, cloud feedbacks etc. Conversely, predictions of atmospheric composition through time, and as different metabolisms emerge, would be extremely useful to researchers across many disciplines and would help to make the paper more impactful.

- Line 333-336. This assumption is confusing and potentially problematic. Why is additional “efficient consumption” of CO by acetogens needed? Isn’t this CO-consumption already included in the ecological model? (except for the MG case). How is the abiotic formate sink parameterized? It appears as though a CO sink is arbitrarily being introduced here to prevent a CO-runaway. But perhaps a CO-runaway is a plausible consequence of an MG-only ecosystem? Further discussion of this CO sink is required.

- The modeling approach adopted in this paper could be extremely valuable to other early Earth researchers, but it would likely be difficult to reproduce from only the description of the model in the paper and supplementary materials. I strongly encourage the authors to make their code publicly available to improve the transparency and reproducibility of their results.

- Line 78: Please define “viable” in this context for the uninitiated. Do the 50% of simulations without viable ecosystems result in population crashing, global glaciation, or both?

- Line 105: Although I suspect photosynthetic life probably did evolve prior to 3.5 Ga (there are putative stromatolites at 3.5 Ga after all), I don’t think an inferred low biomass necessarily establishes this fact. One can imagine an Archean biosphere where burial efficiency is orders of magnitude larger than the modern value (~0.2%). Moreover, large portions of the organic carbon in Precambrian sediments may be detrital (e.g. Daines et al. 2017; Nature Communications). If the authors want to make the argument that even with 100% burial efficiency and incomplete oxidation of organic matter that biomass production 4-7 orders of magnitude below modern is impossible to reconcile with the carbon isotope record, then there should be further quantitative justification. Moreover, if such justification can be provided, then this is an interesting conclusion that should be highlighted more prominently in the paper+abstract.

- Line 138-140: It is unclear how this sentence relates to the previous statement. Please reword.

- Berner (1982; AJS) puts the modern burial efficiency of organic carbon at around 0.2% Most other estimates are also <1% (e.g. 0.3% in Ozaki et al. 2018; Nature Geoscience). I could not find where the 2% value assumed in this study came from in Weiss et al. 2016.

- Additionally, it is unclear how burial efficiency factors into the calculations in the “Dead Biomass” section. Please make this more explicit.

- Lines 28-49 in the supplementary materials. A lot of interesting results are reported here, but the figures supporting them are either not cited or not included. For example, lines 29-32 and lines 38-40. Please either add supporting figures, cite existing figures, or state “not shown” for greater clarity.

**Author rebuttal, first version:**

**Reviewer #1 (Remarks to the Author):**

*The paper by Sauterey et al. models the changes in the climate of the early Earth in response to the evolution of methanogenesis, acetogenesis and methanotrophy. The question is of general interest with important implications on the evolution of life on the early Earth as well as on other planets.*

*While I am not familiar enough with modelling to evaluate that aspect of the paper, the microbiology appears to be sound.*

Response: We thank the Reviewer for her/his positive evaluation

*Figure 2. The meaning of the right hand panel is not clear. For instance, how can biomass production have units of ‘molecules cm-2 s-1’? Molecules of what?*

Response: To clarify this, we define in the legend biomass production as the flux of organic carbon fixation, which is measured in molecules of C cm^-2^ s^-1^.

*Line 29. Rewrite as: “…function preceded the evolution of phototrophs.”*

Response: The abstract has been thoroughly re-written to take all comments from all Reviewers into account. This sentence does not exist anymore.

*Line 41. Substitute “to model” with “when modelling”*

Response: This sentence does not exist anymore.

*Line 50-51. The definition of methanogens is incorrect. Most organisms use “redox potential” as an energy source. The defining character is the electron acceptor, which is CO2 for methanogens. Please rewrite as “(unicellular organisms that couple methane biosynthesis with growth)”*

Response: We agree and rephrased the sentence accordingly (l. 48-49).

*Lines 103-105. This sentence seems to equate biomass production with the amount of standing biomass, which is recorded in the geological record. The amount of standing biomass also depends on the rate of degradation, presumably by heterotrophic prokaryotes. If degradation is slow, the standing biomass can be quite large even with slow production rates.*

Response: Assuming that the carbon cycle is at equilibrium (which should be the case on the time scale considered) the upper bound for organic carbon burial is organic carbon fixation (*i.e.*, biomass production). Krissansen-Totton *et al.* (2015) showed that at 3.6 Gya, the organic fraction of the buried carbon was about ⅔ of what it is today. Even when making the (extreme) assumption that 100% of the biomass produced is rapidly buried, and assuming a constant inorganic burial over time, the maximum organic C burial we obtain would still be about 4-5 times lower than the ~4.10^10^ molecule C cm^-2^ s^-1^ (or 1e13 mol C y^-1^) that would be necessary to explain the isotopic fractionation of C observed in the geological record around 3.6 Gya. We conclude that alternate, much more productive pathways to biomass production, most likely photosynthesis, must have evolved before then. In our revision, we address the question of biomass production in the new section “Biomass production” of the Results, and in the new section “Low biomass production of methanogenic ecosystems” of the Discussion.

*Lines 167-174. Figure 4C seems to state that the probability of glaciation increases with higher fluxes of H2. Since H2 flux only increases MG, why doesn’t it lead to even small methane increases and some warming?*

Response: Increasing the H_2_ outgassing rate causes the atmospheric CH_4_ concentration to reach higher levels, thus causing a larger initial increase of temperature. As the carbon cycle buffers this higher temperature peak, a larger amount of atmospheric CO_2_ is sequestered in the planet’s interior. When the atmospheric CH_4_ is consumed by MT, the system is left with an even greater deficit in CO_2_ and temperature drops even lower. To clarify this mechanism, we enhanced Fig. 5A with two atmospheric and climate trajectories that correspond to two different H_2_ outgassing rates. We also added more details in the main text (l. 261-264).

*Line 198. “other Earths”. Please rewrite. There is only one Earth.*

Response: We now use “Earth analogs” instead of “other Earths”.

**Reviewer #2 (Remarks to the Author):**

*The authors provide results of a coupled biology-planetary model to estimate the influence of a pre-photosynthetic prokaryotic community on planetary habitability. They relate these findings to certain metabolic innovations on earth as well as the potential of chemolithotrophic communities on early earth. This work is novel in that it combines several models, multiple simulations, with statistical evaluations in an effort to describe the earliest carbon cycle on earth. They investigate three different ecosystems, all methane based, and describe the impact each of those would have on planet habitability. This is relevant to the search for life on Mars, of extrasolar planets, as well as our general understanding of how life originated on our planet. The authors also make a strong case that their results give credence or the value of the innovation of photosynthetic metabolism on earth’s habitability. Conceptually, the project is a difficult one. While it is not novel in concept (Kasting et al, referenced in manuscript), it is indeed a novel and vastly improved effort from previous attempts. And it certainly contributes in a substantial way to our understanding that a very primitive community of methane producing and consuming individuals was likely not sufficient to provide climate stability early on. This is satisfying in light of our current assessment of very ancient stromatolitic communities (Allwood, et al). (That is, these early stromatolite fossils were formed through a complex community of organisms whose constituency included photosynthetic members. This corroborates the author’s suggestion that the timing of metabolic innovation was critical in earth’s history early on.) There is application of their model protocols for additional refinements as well as other applications. I would consider the authors’ efforts as valuable for continued research not only in climate and geology but in environmental microbiology. I have heard it said in geology circles that microbial interactions are minimal when regarding the time scale that on which geological change operates. This research offers a different view that as a microbiologist I find satisfying. Not only does it model and fit microbial community dynamics to surface geochemistry and climate despite time scale differences, it posits that it might not even be necessary to have a large microbial biomass present to significantly impact planet habitability. These kinds of insights are the kind that influence thinking across multiple disciplines. Also, there is sufficient detail provided that other researchers could utilize the models and protocols reported here in. I find that modelers of climate and geology are researchers who tend to share code. This report exhibits that to be sure in the method details provided as well as the researchers they have acknowledged.*

Response:

We thank the Reviewer for her/his appreciation of our work.

Our revision echoes the Reviewer’s comments and amplifies some of the conclusions to ensure that they reach across disciplines. Specifically, we made the following changes.

- We put more emphasis on the prediction of low biomass productivity of primitive methane-cycling ecosystems. New sections are devoted to this issue in the Results and in the Discussion. We emphasize that our estimates are lower than reported in previous studies and we explain why (in the section “Biomass production” of the Results and in the section “Low biomass production of primitive methanogenic ecosystems” of the Discussion).
- We added a new analysis of the planetary atmospheric and climatic equilibria corresponding to different metabolic compositions of the biosphere. The analysis is reported in the new section “Long-term effects of metabolic evolution interacting with the carbon cycle” of the Results, illustrated with the new Fig. 4, and discussed in the new section “Atmospheric signature of primitive methane-cycling biospheres” of the Discussion. This analysis yields novel predictions of the general atmospheric signatures of different metabolic states that can be expected as the biosphere evolves and diversifies.
- Our new analysis of the planetary ecosystem equilibria (Fig. 4) provides the backdrop against which we can better evaluate the transient effects of evolutionary innovation on the atmosphere and climate (Fig 5). We emphasize that the propensity of methane-cycling ecosystems to destabilize the early Earth’s climate strongly depends on a tight coupling and dynamical feedback between biological activity and planetary geochemical processes (in particular the abiotic carbon cycle).
- We clarified one of the take-home messages: there is a strong influence of the pace of metabolic evolution on the early Earth’s atmospheric and climatic history. Varying the pace of evolution may not only change the timeline of atmospheric and climatic equilibria; it may reduce climate resilience and cause strong and long transients that keep the planetary system out of equilibrium as the biosphere evolves. This is emphasized in the Results section “Transient climate destabilization by methanotrophy” and in the Discussion section “Climate destabilization by life-planetary feedbacks”.

**Reviewer #3 (Remarks to the Author):**

*This paper presents a new approach to understanding biogeochemical cycles on the early Earth. A detailed model of chemolithoautotrophic ecosystems is developed that captures microbial population dynamics by considering the thermodynamic and kinetic limitations to cell growth. This ecosystem model is coupled to parameterized climate, carbon cycle, and photochemical models to investigate the ways in which biological feedbacks and evolutionary innovations could modulate planetary habitability. The key findings are that (i) biological methane cycling by non-photosynthetic ecosystems can strongly affect atmospheric composition despite total biomass being low, (ii) biological methane cycling has an anti-Gaian tendency to amplify geologically driven temperature perturbations, and (iii) the evolution of methanotrophy may have resulted in dramatic global cooling.*

*The novel modeling approach in this paper is impressive. By explicitly incorporating the thermodynamic and kinetic limitations on metabolism and self-consistently modeling cell growth, catabolism, anabolism, and cell death, the ecosystem model seemingly has the potential to explore biogeochemical interactions much more rigorously than has been attempted previously. The authors ought to be commended for this innovative approach.*

*I feel as though the paper is incomplete in its current form. Although I can see the potential of this kind of detailed modeling, the paper does not clearly demonstrate how and why their model makes different predictions to previous models of methane-cycling ecosystems. The significant complexity added via ecosystem modeling requires further justification.*

*Moreover, the novelty of the main results needs to be better highlighted and quantified. The idea that methane cycling in non-photosynthetic ecosystems strongly affects atmospheric composition, and therefore climate, is not new (e.g. Kharecha et al. 2005). Similarly, the idea that evolutionary innovations such as the emergence of methanotrophy could trigger glaciations is fairly unsurprising. This paper requires major revisions to better highlight novel conclusions and justify the modeling approach.*

*Methodologies and assumptions also need to be clarified at several points in the manuscript.*

Response: We thank the Reviewer for her/his thorough and insightful comments. We heeded all of the Reviewer’s very helpful suggestions, with the aim of better justifying our modeling approach and emphasizing the novelty of our findings.

Our approach advances previous attempts at describing the interaction between primitive biospheres and the atmosphere and climate of the early Earth (e.g., Kasting *et al*. 2001, Kharecha *et al.* 2005, Ozaki *et al.* 2018) by dynamically coupling biological activity and the planet’s surface geochemistry. This is made possible at the level of individual microbial cells by explicitly modelling the efficiency of the metabolic coupling between energy acquisition (anabolism) and biomass production (catabolism). We use our new model to

1. Predict biomass productivity of primitive, non-photosynthetic biospheres, taking into account the decreasing metabolic efficiency of catabolism-anabolism coupling as the biosphere consumes the bioavailable redox potential of the atmosphere. Our predictions are significantly lower than previous estimates. We now put more emphasis on this result and its implications regarding the timeline of metabolic evolution on Earth, especially the evolution of photosynthetic life (more on this in our response to comment 1 below).
2. Close the feedback loop between atmosphere, climate and biological activity. This yields new insights into the short-term and long-term effects of metabolic evolution on the atmosphere and climate. These results go beyond the scope of previous models that only captured the thermodynamic effect of temperature on metabolism and did not monitor feedbacks on the atmospheric composition and climate.
3. Identify atmospheric signatures of a metabolically evolving ecosystem that can distinguish pre-photosynthetic methane-based biospheres from a life-less planetary state and are robust to the atmospheric and climatic instability and transients caused by evolutionary innovations.
4. Evaluate the magnitude and duration of such evolutionarily induced transients in terms of atmospheric composition and climate and their implication for the evolution of the planet’s habitability.

Following the Reviewer’s suggestion, we performed a complete analysis of the atmospheric and climate equilibrium states in response to plausible alternate evolutionary trajectories. The new results make a significant addition to our manuscript (new Fig. 4, new Results section “Long-term effects of metabolic evolution interacting with the carbon cycle”, and new Discussion section “Climate destabilization by life-planetary feedbacks”). We highlighted the implications in terms of the atmospheric signature of the biosphere at different stages of its evolutionary history, which would apply to life on the early Earth as well as putative life on Earth analogs. Furthermore, our new analysis of the planetary atmosphere and climate equilibrium with biological activity provides a clear backdrop against which the climatic transients triggered by evolutionary metabolic transitions can be presented and discussed.

Part of the revised Introduction (l 62-74) highlights why improving on previous approaches in the way we did was necessary to gain new, significant insights into the global environmental feedback driving the coevolution of life and its planetary environment. We reorganized the Abstract, Introduction, Results, and we created a separate Discussion section, in order to make our main conclusions more impactful and more accessible to readers of diverse scientific backgrounds. Hereafter we explain the changes we made in more detail. We numbered the comments for ease of cross-referencing.

*Specific comments:*

1. *The abstract states that “How primitive, non-photosynthetic ecosystems influenced the early habitability of Earth is poorly understood. A full understanding of this issue requires models that couple the ecological function and biological evolution of chemolithotrophic ecosystems and their interactions with the planet surface geochemistry and climate.” The paper ought to do more to convince the reader that these ecological models provide new insights compared to previous models where ecosystems are represented by net reactions added to photochemical models (e.g. Kharecha et al. 2005, Geobiology; Schwieterman et al. 2019, ApJ). For example, how do biomass production outputs in Fig. 2 compare to those for the same metabolisms in Kharecha et al. 2005? An explicit graphical comparison would be helpful. It looks like the estimates in this study are considerably lower. Why is this the case?*

Response: A graphical comparison between our predictions of biomass productivity (and biogenic CH_4_ emission) and Kharecha *et al.*’s (2005) is shown in Fig. 2 (dashed lines). In our revision we provide more detail to explain why our results differ so markedly. This is done in a dedicated Results section “Biomass production”, that we developed and expanded from results and supplementary material in the previous version of the manuscript. A major difference with previous work is that our model relaxes the assumption that the yield in terms of biomass production per molecule of electron donor consumed is fixed and independent of the state of the environment. Instead we use thermodynamics constraints to model how the efficiency of the coupling between energy acquisition (catabolism) and biomass production (anabolism) decreases as the biosphere progressively depletes metabolic substrates. Our results highlight that improved predictions of ecosystem function and its effect on the atmosphere and climate requires to model the dynamical and reciprocal interaction of biological activity and surface geochemistry. Key to this feedback is how biologically-driven environmental changes (in substrate availability and temperature) modifies the coupling between energy acquisition and biomass production. In our revision, this critical feature of the model is presented more prominently, and we emphasize the major difference that the full feedback model makes on predictions of biomass production, in the Introduction (l. 62-74) and most importantly in the opening paragraphs of the new Discussion. .

2. *The main text assumes that methanotrophy evolved shortly after methanogenesis, but it could be argued that methanotrophy probably didn’t emerge until after oxygenic photosynthesis due to lack of viable oxidants in the Archean ocean. There is some discussion of this issue in the supplementary materials, but I think this needs to be explored in the main text since it underlies Fig. 4.*

Response: There is indeed a consensus that aerobic methanotrophy evolved contemporaneously with oxygenic photosynthesis (Moore *et al.* 2018, Battistuzzi *et al.* 2005). In contrast, the timing of the origin of anaerobic oxidation of methane (AOM), that is linked to the reduction of nitrate, nitrite, or sulfate, is not well resolved. Since AOM proceeds essentially as a reversal of methanogenesis in terms of the enzyme involved, it has been suggested that AOM could have evolved soon after methanogenesis (Knittel and Boetius 2009, Nitschke and Russel 2019). The evolution of AOM prior to photosynthesis also appears to be compatible with the sulfur isotopic fractionation record (l 278-281 and 363-365, see also point 3 below). In our work we adopted these views to explore the dynamics and planetary influence of a biosphere that could evolve towards fully closing a methane cycle before the evolution of photosynthesis. We clarified this point in the (new) Discussion, section “Climate destabilization by life-planetary feedbacks”, where we integrated material formerly presented in the Supplementary information. And even though we present results for pre-photosynthetic evolutionary sequences that end with the evolution of methanotrophy, we emphasize that alternate timing for the evolution of methanotrophs leads to results similar to those displayed in Fig. 4 (as methanotrophy has very little effect on the equilibrium state of the planet) and 5 (across simulations, the evolution of methanotrophy triggers the sharp climatic response described in the main text regardless of the metabolic composition of the methanogenic biosphere, results not shown).

3. *On a related note, I am unsure as to whether this paper’s estimates of the Archean sulfate reservoir are reasonable. For example, Crowe et al. (2014; Science) estimate Archean sulfate concentrations to be <2.5 micromolar (c.f. 4 mM assumed in this study). Habicht et al. (2002; Science) estimate concentrations of <0.2 mM. Admittedly, these estimates assume some form of biological sulfate reduction, but microbial sulfate reduction could conceivably use reductants other than methane.*

*The 4 mM oceanic concentration used in this study seems to come from a 7.2e12 L/yr flux of water through hydrothermal systems, and an assumption that all sulfate is removed in these high-temperature systems. But this is not necessarily the only significant sulfate sink in the reducing Archean environment. Low-temperature aqueous reactions and/or evaporative sinks could contribute, leading to a smaller sulfate reservoir.*

Response: Indeed, the pre-methanotrophy 4mM sulfate oceanic concentration corresponds to the balance between sulfate source (rainout) and sink (hydrothermal circulation). We agree that other sinks could contribute, leading to a smaller sulfate concentration. Those 4mM in sulfate concentration corresponds to the removal of ~3e4 ppm of CH_4_, assuming instantaneous CH_4_ reduction by methanotrophs. This is about 10 times higher than the upper limit for CH_4_ atmospheric concentration predicted by our ecosystem model. Consequently, even with a sulfate concentration 10 times lower (hence with a sulfate sink 10 times faster), the atmospheric CH_4_ would still plummet after the emergence of methanotrophs. However, as explained in the main text l. 267-282, this would be a transient state. The biological methane flux is higher than the sulfate flux in all of our simulations. Once methanotrophs become sulfate limited, an equilibrium state is reached with high CH_4_ atmospheric concentrations and very low H_2_SO_4_ oceanic concentrations, below the µM. Such concentrations are compatible with constraints from Habicht *et al.* (2002) and Crow *et al.* (2014). Given the constraints from Crow *et al.* (2014) (sulfate oceanic concentration <2.5 µM), either methanotrophy has no role to play in setting oceanic sulfate concentration and we underestimate the abiotic sulfate sink by 3 orders of magnitude or, as our result suggest, methanotrophs were present during the Archean and efficiently consumed the available sulfate. This is now explained in the new Results section “Transient climate destabilization by methanotrophy” (last paragraph).

To address this point more thoroughly, we conducted a sensitivity analysis on the value of the sulfate source (which is equivalent to testing their sensitivity to the value of the sulfate sink) by varying it by two orders of magnitude (see Table 1). For the reason given above, we find that our results are basically insensitive to this parameter.

4. *The paper implicitly assumes that life is not nutrient limited. This assumption needs to be made explicit and justified, with appropriate caveats. Some estimates of Archean phosphorus abundances in the ocean suggest concentrations 3-5 orders of magnitude less than in the modern ocean (e.g. Kipp and Stueken 2017; Science Advances, and references therein). This overlaps with the Archean biomass estimates in this paper (4-7 orders of magnitude lower than modern), and so the possibility of phosphorus limited non-photosynthetic biospheres cannot be excluded. Nitrogen limitation presumably also cannot be ruled out unless it has been established that biological nitrogen fixation predates methanogenesis and acetogenesis.*

Response: In their seminal paper, Kharecha *et al.* (2005) discuss the assumption that biomass production by primitive biospheres should have been limited by the availability in electron donors rather than by nutrients such as N and P. More recently and more extensively, Ward *et al.* (2019) evaluated the most likely limitation to biomass production prior to the evolution of oxygenic photosynthesis. By assuming a fixed yield for biomass production per molecules of electron donor consumed (as Kharecha *et al.* (2005) did), they found that the N and P supplies were most likely sufficient during the Archean for electron donors to be limiting. This turns out to be even more likely given our finding of a biomass productivity lower than levels estimated by Kharecha *et al.* (2005) and Ward *et al.* (2019).

Kipp and Stuekken (2017) discussed P limitation as potentially due to the limited availability of oxidative species and inefficient remineralization of organic P as a consequence. This is an interesting hypothesis, especially for systems in which the rate of P fixation in biomass largely exceeds the rate of P weathering, in which case biomass production indeed strongly depends on remineralization efficiency. This does not, however, apply to our system. Indeed, based on our highest estimates of biomass productivity and applying the C:P Redfield ratio of 1:106, we predict very low P fixation rates, about 3 times lower than the modern P weathering rate (~ 7 10^10^ mol y^-1^), and potentially even lower relative to Archean P weathering, which may have been more efficient than modern weathering (Hao *et al.* 2017). Therefore, the main P supply to surface ocean would have been weathering regardless of remineralization efficiency, and under such circumstance, Ward *et al.*’s (2019) analysis remains valid.

In our revision, we addressed the important question of biomass production and nutrient limitation in the new Discussion section “Low biomass production of primitive methanogenic ecosystems”.

5. *Some discussion of the likely order of emergence of methanogenesis vs acetogenesis/acetotrophy would be helpful. Would a world with methanogens but no acetogens persist for very long? Presumably the CO-accumulation that results from a methanogen-only ecosystem would be a strong evolutionary driver for CO-consuming acetogens? What do LUCA reconstructions have to say on the subject?*

Response: We are responding to points 5 and 6 together. Please see our response to point 6 below.

6. *On a related note, could the model outputs from this study be used to make some quantitative, testable predictions about the Archean atmosphere’s composition through time? Recent studies have helped constrain the hydrogen and methane abundances of the early Earth (e.g. Kadoya and Catling, 2019, GCA; Zahnle et al. 2019, GCA). More precise proxies will surely follow. In my mind, quantitative predictions of atmospheric composition would be more useful than the climate perturbation/glaciation calculations in Fig. 4 since the latter will be modulated by uncertain paleogeography, cloud feedbacks etc. Conversely, predictions of atmospheric composition through time, and as different metabolisms emerge, would be extremely useful to researchers across many disciplines and would help to make the paper more impactful.*

Response: We embraced the Reviewer’s suggestions and performed a new set of analyses to obtain the predictions that s/he was calling for. Starting with point 5, phylogenetic analyses suggest that both methanogenesis and acetogenesis are good candidates for the most ancestral metabolic pathways (e.g., Weiss *et al.* 2016, Martin *et al*. 2016, Havig et al. 2017). In Fig. 4 and corresponding text (new Results section “Long-term effects of metabolic evolution interacting with the carbon cycle”), we show how metabolic evolution may have influenced the state of the planetary atmosphere and climate. We consider all pre-methanotrophy metabolic communities that are viable (*i.e.*, in which all the metabolic types can sustain population growth), given plausible environmental conditions for the Archean (drawn in uniform priors based on the literature). These metabolic communities are: AG, MG, AG+AT, MG+AG, MG+AG+AT, MG+AG+AT. We consider all possible evolutionary transitions among them (Fig. 4A), and assume that metabolic diversification culminates with the evolution of methanotrophs. All these pathways are plausible based on available phylogenetic information (Battisttuzzi *et al.* 2005, Weiss *et al*. 2016, Martin *et al*. 2016, Marin *et al*. 2017) (see also our response to point 2 regarding the evolution of methanotrophy). We then conduct an equilibrium analysis of the planetary ecosystem in each case, in order to evaluate the influence of each biosphere on the planet’s atmosphere and climate (Fig. 4B-E). This provides posterior distributions of atmospheric composition (Fig. 4B-D) given a specific metabolic composition of the biosphere. These new results are presented in Fig. 4. Together with Fig. 5 (formerly Fig. 4), they show that evolutionary innovations that have no effect on the system equilibrium may yet cause strong climatic transients. This is discussed in the new Discussion section “Climate destabilization by life-planetary feedbacks”.

7. *Line 333-336. This assumption is confusing and potentially problematic. Why is additional “efficient consumption” of CO by acetogens needed? Isn’t this CO-consumption already included in the ecological model? (except for the MG case). How is the abiotic formate sink parameterized? It appears as though a CO sink is arbitrarily being introduced here to prevent a CO-runaway. But perhaps a CO-runaway is a plausible consequence of an MG-only ecosystem? Further discussion of this CO sink is required.*

Response: Our earlier phrasing was ambiguous indeed. The photochemical model is not directly coupled to the ecosystem model. We compute the chemical destruction/production rates for a grid of atmospheric compositions (CO2, CH4 and H2) at steady state. CO is an outcome of the atmospheric photochemical reactions. Without sinks, the system would experience a CO-runaway scenario that would prevent the model from converging. However, acetogens, when they are present in the biosphere, remove CO from the atmosphere at a fast rate, close to the diffusion limit (~10^-4^ cm s^-1^). In addition to this biotic sink, similarly to Kharecha *et al.* (2005) and Hu *et al.* (2020 ), there is an abiotic sink of CO in the model is caused by the CO-consuming production of formate in the ocean, with a CO deposition velocity of 10^-8^ cm s^-1^. Together these two sinks ensure convergence of our model in all the scenarios we explore. This is now explained in more detail in the “photochemistry” section of the Methods (l. 542-547).

8. *The modeling approach adopted in this paper could be extremely valuable to other early Earth researchers, but it would likely be difficult to reproduce from only the description of the model in the paper and supplementary materials. I strongly encourage the authors to make their code publicly available to improve the transparency and reproducibility of their results.*

Response: Given that this research program is on-going and more publications using this model and code are in preparation, we followed the practice of other teams working in the field and offer to deposit the code on a private Gitlab repository and provide access to it "upon reasonable request" to the corresponding author (as in Ozaki *et al.* 2019 *Nature Communications*).

9. *Line 78: Please define “viable” in this context for the uninitiated. Do the 50% of simulations without viable ecosystems result in population crashing, global glaciation, or both?*

Response: In ecology, a modelled population is “viable” if the model of population biomass dynamics possesses a positive equilibrium (or an attractor in the positive quadrant, bounded away from zero). We rephrased to make this clearer (l. 97-99).

10. *Line 105: Although I suspect photosynthetic life probably did evolve prior to 3.5 Ga (there are putative stromatolites at 3.5 Ga after all), I don’t think an inferred low biomass necessarily establishes this fact. One can imagine an Archean biosphere where burial efficiency is orders of magnitude larger than the modern value (~0.2%). Moreover, large portions of the organic carbon in Precambrian sediments may be detrital (e.g. Daines et al. 2017; Nature Communications). If the authors want to make the argument that even with 100% burial efficiency and incomplete oxidation of organic matter that biomass production 4-7 orders of magnitude below modern is impossible to reconcile with the carbon isotope record, then there should be further quantitative justification. Moreover, if such justification can be provided, then this is an interesting conclusion that should be highlighted more prominently in the paper+abstract.*

Response: A similar point was raised by Reviewer 1, and the same response applies here. Assuming that the carbon cycle is at equilibrium (which should be the case on the timescale that we consider) the upper bound for organic carbon burial is organic carbon fixation (biomass production). Krissansen-Totton *et al.* (2015) showed that at 3.6 Gya, the organic fraction of the buried carbon was about ⅔ of what it is today. Even when making the (unrealistic) assumption that 100% of the biomass produced is rapidly buried, and assuming that inorganic burial is constant over time, the maximum organic C burial we obtain would still be about 4-5 times lower than the ~4.10^10^ molecule C cm^-2^ s^-1^ (or 1e13 mol C y^-1^) requiredy to explain the isotopic fractionation of C observed in the geological records at 3.6 Gya. We therefore conclude that alternative pathways to biomass production, most likely photosynthetic ones, should have evolved before then. Following the Reviewer’s advice, we highlighted this result more prominently, in the Abstract, in a new Results section “Biomass production”, and in a new Discussion section “Low biomass production of primitive methanogenic ecosystems”.

11. *Line 138-140: It is unclear how this sentence relates to the previous statement. Please reword.*

Response: We rephrased for clarity (l. 152-154): we find that most of the time, the availability of electron donors rather than the formation of organic haze would have limited climate warming by methanogenesis.

12. *Berner (1982; AJS) puts the modern burial efficiency of organic carbon at around 0.2% Most other estimates are also <1% (e.g. 0.3% in Ozaki et al. 2018; Nature Geoscience). I could not find where the 2% value assumed in this study came from in Weiss et al. 2016.*

Response: 2% is the upper limit for burial efficiency considered in Kharecha *et al.* (2005). We consider the modern value of burial efficiency of 0.2% as our lower bound, and take 100% as the upper bound (see also our responses to points 10 and 13).

13. *Additionally, it is unclear how burial efficiency factors into the calculations in the “Dead Biomass” section. Please make this more explicit.*

Response: We rewrote this section of the Methods to make it clearer (l 498-519). To optimize computation time, we did not model biomass recycling explicitly; instead we assumed that the dead biomass accumulates in the ocean. We verified that this accumulation does not affect our predictions in terms of equilibrium atmospheric composition by introducing biological recycling in a subset of simulations; we found that the flux of biomass production was so small that dead biomass remineralization had no impact on the composition of the atmosphere (l. 505-508). This is also illustrated by the fact that the biomass production, or lack thereof, does not alter the global redox balance of the atmosphere (Supplementary Results and Discussion, l. 508-511; Fig. S12). Furthermore since our biological model uses the intracellular concentration of biomass to evaluate metabolic thermodynamics, the accumulation of dead biomass does not affect metabolic efficiencies. As in most previous studies, we evaluate burial as a fraction of biomass production. We then consider 0.2 % and 100 % as lower and upper bounds for the fraction of biomass buried.

14. *Lines 28-49 in the supplementary materials. A lot of interesting results are reported here, but the figures supporting them are either not cited or not included. For example, lines 29-32 and lines 38-40. Please either add supporting figures, cite existing figures, or state “not shown” for greater clarity.*

Response: We appreciate the Reviewer’s patience with this. We scrutinized our supplementary material to make sure that results in the text were properly supported by the appropriate (and correctly referenced) figures. Also, we moved a substantial portion of the supporting discussion into the new Discussion in the main text, where the important points raised by the Reviewer, regarding low biomass production and its implications, ocean sulfate stocks and methanotrophy, are now addressed more prominently.

**References**

Battistuzzi, Fabia U., Andreia Feijao, and S. Blair Hedges. "A genomic timescale of prokaryote evolution: insights into the origin of methanogenesis, phototrophy, and the colonization of land." *BMC evolutionary biology* 4.1 (2004): 44.

Crowe, Sean A., et al. "Sulfate was a trace constituent of Archean seawater." *Science* 346.6210 (2014): 735-739.

Habicht, Kirsten S., et al. "Calibration of sulfate levels in the Archean ocean." *Science* 298.5602 (2002): 2372-2374.

Hao, Jihua, Dimitri A. Sverjensky, and Robert M. Hazen. "Mobility of nutrients and trace metals during weathering in the late Archean." *Earth and Planetary Science Letters* 471 (2017): 148-159.

Havig, Jeff R., et al. "Sulfur and carbon isotopic evidence for metabolic pathway evolution and a four-stepped Earth system progression across the Archean and Paleoproterozoic." *Earth-Science Reviews* 174 (2017): 1-21.

Hu, Renyu, Luke Peterson, and Eric T. Wolf. "O2-and CO-rich Atmospheres for Potentially Habitable Environments on TRAPPIST-1 Planets." *The Astrophysical Journal* 888.2 (2020): 122.

Kasting, James F., Alexander A. Pavlov, and Janet L. Siefert. "A coupled ecosystem-climate model for predicting the methane concentration in the Archean atmosphere." *Origins of Life and Evolution of the Biosphere* 31.3 (2001): 271-285.

Kharecha, P., James Kasting, and J. Siefert. "A coupled atmosphere–ecosystem model of the early Archean Earth." *Geobiology* 3.2 (2005): 53-76.

Kipp, Michael A., and Eva E. Stüeken. "Biomass recycling and Earth’s early phosphorus cycle." *Science advances* 3.11 (2017): eaao4795.

Knittel, Katrin, and Antje Boetius. "Anaerobic oxidation of methane: progress with an unknown process." *Annual review of microbiology* 63 (2009): 311-334.

Krissansen-Totton, Joshua, Roger Buick, and David C. Catling. "A statistical analysis of the carbon isotope record from the Archean to Phanerozoic and implications for the rise of oxygen." *American Journal of Science* 315.4 (2015): 275-316.

Marin, Julie, et al. "The timetree of prokaryotes: new insights into their evolution and speciation." *Molecular biology and evolution* 34.2 (2016): 437-446.

Martin, William F., and Filipa L. Sousa. "Early microbial evolution: the age of anaerobes." *Cold Spring Harbor perspectives in biology* 8.2 (2016): a018127.

Moore, Eli K., et al. "Metal availability and the expanding network of microbial metabolisms in the Archaean eon." *Nature Geoscience* 10.9 (2017): 629-636.

Nitschke, Wolfgang, and Michael J. Russell. "Beating the acetyl coenzyme A-pathway to the origin of life." *Philosophical Transactions of the Royal Society B: Biological Sciences* 368.1622 (2013): 20120258.

Ozaki, Kazumi, et al. "Effects of primitive photosynthesis on Earth’s early climate system." *Nature Geoscience* 11.1 (2018): 55-59.

Ward, Lewis M., Birger Rasmussen, and Woodward W. Fischer. "Primary productivity was limited by electron donors prior to the advent of oxygenic photosynthesis." *Journal of Geophysical Research: Biogeosciences* 124.2 (2019): 211-226.

Weiss, Madeline C., et al. "The physiology and habitat of the last universal common ancestor." *Nature microbiology* 1.9 (2016): 1-8.

**Reviewer comments, second version:**

REVIEWERS' COMMENTS:

Reviewer #1 (Remarks to the Author):

My comments were satisfactorily addressed in the revision.


Reviewer #2 (Remarks to the Author):

The authors have accurately and thoroughly answered the reviewers questions. Its a great paper.


Reviewer #3 (Remarks to the Author):

The authors have thoroughly addressed all my comments in the revised manuscript. The addition of Fig. 4 and associated discussion will be very helpful for researchers who are interested in the composition of the Archean/Hadean atmosphere. More generally, the novel modeling framework presented in this study constitutes a major advancement in our understanding of the co-evolution of life and the environment. I recommend the paper be promptly accepted for publication.

**Author rebuttal, second version:**

**Reviewer comments, third version:**

**Author rebuttal, third version:**
